# SARS-CoV-2 infection is associated with hypothalamic orexin suppression and persistent cortical NeuN attenuation

**DOI:** 10.1186/s12974-026-03842-y

**Published:** 2026-05-05

**Authors:** Gun Young Yoon, Young Cheul Chung, Ji Hyun Choi, Yun Ha, Se Yeon Seo, Keun Bon Ku, Do Yeon Kim, Woo Yeon Hwang, Gi Uk Jeong, Dae-Gyun Ahn, Kyun-Do Kim, Je-Keun Rhee, Won-Ho Shin, Young-Chan Kwon

**Affiliations:** 1 Center for Infectious Diseases Vaccine and Diagnosis Innovation (CEVI), Korea Research Institute of Chemical Technology, Daejeon, 34114 Republic of Korea; 2 Division of Advanced Predictive Research, Korea Institute of Toxicology, Daejeon, 34114 Republic of Korea; 3 Department of Bioinformatics & Life Science, Soongsil University, Seoul, 06978 Republic of Korea; 4 College of Pharmacy, Korea University, Sejong, 30019 Republic of Korea; 5 Department of Microbiology, Sungkyunkwan University School of Medicine, Suwon, 16419 Republic of Korea; 6 AI-Bio Convergence Research Institute, Soongsil University, Seoul, 06978 Republic of Korea; 7 Medical Chemistry and Pharmacology, University of Science and Technology (UST), Daejeon, 34114 Republic of Korea; 8 Human and Environmental Toxicology, University of Science and Technology (UST), Daejeon, 34114 Republic of Korea

**Keywords:** Long COVID, NeuN, *Hcrt* (Orexin), SARS-CoV-2, Neurological pathogenesis

## Abstract

Long COVID frequently presents with persistent neurological symptoms, including cognitive impairment, fatigue, and sleep disturbances; however, its underlying mechanisms remain unclear. Here, we show that SARS-CoV-2 infection induces lasting cortical neuronal injury and hypothalamic orexin (hypocretin) dysfunction in vivo. In K18-hACE2 and wild-type BALB/c mice, viral RNA persisted in the brain and coincided with focal loss of Neuronal Nuclei (NeuN)-positive cortical neurons beyond acute infection. SARS-CoV-2, but not the influenza A virus, triggered rapid and sustained suppression of hypothalamic orexin expression, defining a virus-specific neuropathological signature. Considering the downregulation of orexin and focal cortical NeuN attenuation, we found that exogenous orexin-A/B supplementation increased NeuN abundance in vitro and in vivo under the tested conditions. Overall, these findings identify the orexin system as a candidate neural vulnerability to SARS-CoV-2 and suggest that orexinergic dysfunction may contribute to the neurological manifestations of Long COVID.

**Supplementary Information:**

The online version contains supplementary material available at 10.1186/s12974-026-03842-y.

## Introduction

Long COVID, defined as persistent symptoms lasting beyond four weeks after acute SARS-CoV-2 infection, affects approximately 10–30% of convalescent individuals [1, 2]. Neurological and neuropsychiatric manifestations, such as cognitive impairment, sleep disturbance, fatigue, and attention deficits, represent some of the most debilitating outcomes documented across longitudinal cohorts [3–5]. Large-scale neuroimaging studies have revealed that SARS-CoV-2 infection induces long-lasting alterations in cerebral structure and function, including progressive cortical thinning, grey matter loss, and disruption of functional connectivity [6–8]. These changes correlate with persistent cognitive symptoms, indicating sustained disruption of neural circuits beyond the acute phase [5–7].

Several mechanisms have been proposed to explain these effects. Neuropathological studies have revealed viral RNA and proteins in neurons, astrocytes, and microglia, confirming central nervous system (CNS) tropism [9–11]. Robust systemic immune activation is associated with cognitive symptoms and blood–brain barrier breakdown [9, 12]. However, the current neuroinflammatory models do not fully account for these key observations. Neurological sequelae often persist after overt inflammatory signatures wane, and all neurotropic viruses do not produce the same phenotype despite comparable immune activation [13, 14]. This dissociation suggests that SARS-CoV-2 targets specific neural populations or homeostatic circuits whose dysfunction sustains neurological deficits.

Among these circuits, orexin (hypocretin), a neuropeptide that couples vigilance with energy balance, is produced by a small, anatomically restricted population of lateral hypothalamic neurons that nevertheless exerts outsized control over wakefulness and attention [15–17]. Selective loss of orexin neurons causes narcolepsy type 1, a disorder that shares key features with Long COVID, including sleep fragmentation and cognitive dysfunction [18, 19]. Beyond regulating arousal, orexin promotes neuronal survival under oxidative stress [20] and limits neuroinflammation [21]. However, whether orexin signalling directly regulates cortical neuronal integrity remains unclear. Given the established roles of the orexin system in homeostatic control and neuronal protection [15–17, 21, 22], we hypothesised that SARS-CoV-2 infection compromises this critical network, potentially reducing circuit resilience [23, 24].

In this study, we identified two convergent in vivo signatures of SARS-CoV-2 neuropathology: focal, heterogeneous loss of cortical NeuN immunoreactivity persisting beyond the acute phase, and selective suppression of hypothalamic hypocretin/orexin (*Hcrt*) expression during early infection. Using comparative viral models, we showed that this specific combination was intrinsic to SARS-CoV-2 and was not recapitulated by the influenza A virus, despite showing comparable neuroinvasion. Furthermore, administering recombinant orexin-A/B partially upregulated NeuN expression during infection. These findings identify the orexinergic system as a selective target of SARS-CoV-2 and suggest that disrupted orexin signalling may compromise cortical neuronal integrity during infection, thereby providing a new framework for understanding the persistent neurological consequences of COVID-19.

## Results

### Persistent cortical neuronal dysfunction and prolonged brain viral RNA in SARS-CoV-2 infected K18-hACE2 mice

**Fig. 1:**
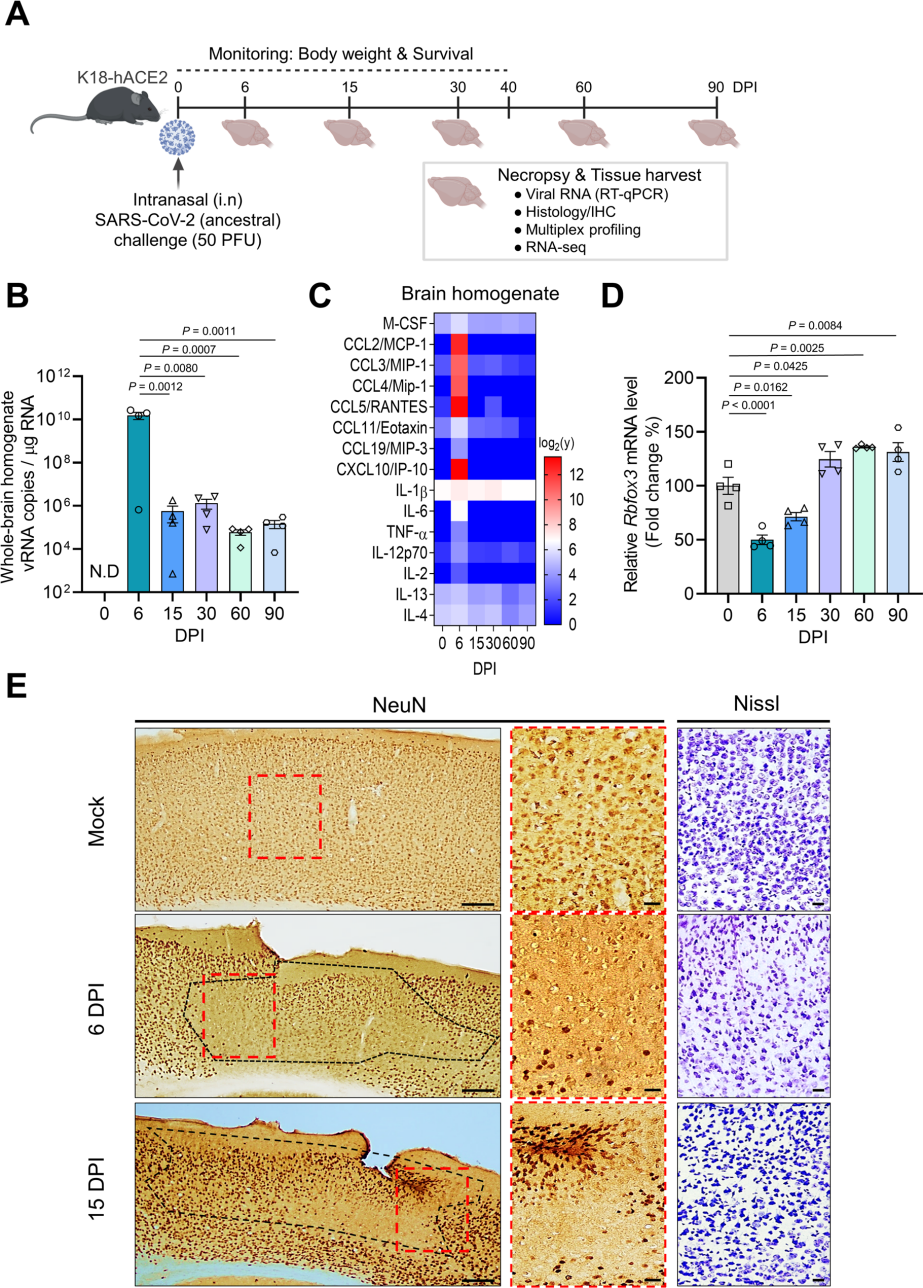
SARS-CoV-2 infection causes transient *Rbfox3* transcriptional suppression but persistent focal loss of cortical NeuN immunoreactivity in K18-hACE2 mice. **A** Schematic of the experimental timeline. K18-hACE2 mice were intranasally challenged with 50 plaque forming units (PFU) of ancestral SARS-CoV-2 and monitored for up to 90 days post-infection (dpi). Tissues were collected at the indicated time points for viral load analysis, multiplex cytokine profiling, histopathology, and bulk RNA sequencing (n = 3–5 mice per group per time point). **B** Time-course quantification of viral RNA burden in whole-brain homogenates using RT-qPCR, presented as viral RNA copies per µg of total RNA. **C** Heat map of cytokine and chemokine protein levels in brain lysates at the indicated time points, measured using a multiplex array. The data represent log2-transformed concentrations (pg mg⁻¹ protein). Values < LOD were set to 1.0 for log transformation. **D** Time-course quantification of *Rbfox3* (NeuN) mRNA expression in whole-brain lysates, presented as fold change relative to mock controls. **E** Representative NeuN immunohistochemistry (left) and Nissl staining (right) in the cerebral cortex of mock- and SARS-CoV-2-infected mice at 6 and 15 dpi. Red dashed boxes indicate high-magnification views of the regions with reduced NeuN immunoreactivity. Black dashed lines demarcate areas with reduced NeuN immunoreactivity. Scale bars, 100 μm (low magnification) and 25 μm (high magnification). Later histological time points demonstrating persistence of the cortical NeuN-low phenotype are shown in Supplementary Fig. 4. Representative images from *n* = 3–5 independent biological replicates are shown. Statistical significance for (**B**) and (**D**) was determined using a one-way ANOVA with Dunnett’s multiple comparisons test. The exact *P* values are indicated; N.D., not detected

K18-hACE2 mice are an established model for severe COVID-19, recapitulating the key features of CNS involvement, including viral neuroinvasion and neuroinflammation [25, 26]. We first confirmed the brain permissiveness following intranasal infection with a lethal dose (2 × 10^4^ PFU) of SARS-CoV-2, which led to robust NP detection in the olfactory bulb and cerebral cortex at 6 days post-infection (dpi) (Supplementary Fig. 1). To model long COVID, we employed a low-dose (50 PFU) infection paradigm, enabling long-term assessment for up to 90 dpi (Fig. 1A). Infected mice showed transient weight loss that was recovered by 7–8 dpi, with survival stabilising at approximately 60% (Supplementary Fig. 2A-B). To define the viral tropism and clearance kinetics, we quantified the viral burden across tissues. At 6 dpi, viral RNA was abundant in peripheral tissues, with high levels in the lung, nasal turbinate, and eye, but was undetectable in the testis, and subsequently declined to near-baseline levels by 60–90 dpi (Supplementary Fig. 2C-F). In contrast, viral RNA in the brain peaked at 6 dpi and remained moderately detectable through 90 dpi, indicating prolonged persistence in the brain relative to that in peripheral tissues (Fig. 1B). Given that SARS-CoV-2 infection induces neuroinflammation in this model [27], we analysed the cytokine and chemokine levels in the brain tissue. Multiplex cytokine profiling revealed robust induction of pro-inflammatory mediators—including CCL2, CXCL10, IL-6, and TNF-α—in the brain at 6 dpi, coinciding with the peak viral load (Fig. 1C and Supplementary Fig. 3). This induction was transient, with most analytes returning toward the baseline by 15–30 dpi; however, IL-1β levels remained modestly elevated through 30 dpi before declining. Despite the resolution of acute cytokine induction, we observed persistent neuronal alterations. Transcriptional analysis of the mature neuronal marker *Rbfox3* (NeuN) in whole-brain tissue showed transient downregulation at 6 dpi, which was recovered by 30 dpi (Fig. 1D). In contrast, immunohistochemical analysis revealed persistent NeuN-depleted regions in the cerebral cortex through at least 60 dpi, but not in the olfactory bulb or hippocampus (Supplementary Fig. 4). Distinct areas of reduced NeuN immunoreactivity—, demarcated by black dashed lines—, emerged acutely at 6–15 dpi (Fig. 1E). High-magnification views of these regions (red dashed boxes) confirmed a profound reduction in NeuN immunoreactivity compared with that in mock controls. The extent of these focal cortical NeuN-depleted regions was further assessed by semi-quantitative area analysis (Supplementary Fig. 5). However, Nissl staining of the adjacent cortical areas revealed that neuronal cell density remained largely intact, although the neurons often exhibited a shrunken or condensed morphology (Fig. 1E). Interestingly, the distribution of this phenotype was spatially heterogeneous; these “patchy” areas appeared in stochastic, multifocal locations across individual mice rather than targeting a fixed cortical subregion (Supplementary Fig. 4B and C). We did not observe a consistent restriction of these NeuN-low regions to a single cortical subregion or laminar compartment across animals. Together, these data indicate that SARS-CoV-2 infection is associated with sustained, spatially heterogeneous reductions in cortical NeuN immunoreactivity, which persist despite the resolution of acute cytokine induction.

### Hypothalamic hypocretin/orexin signalling is suppressed during SARS-CoV-2 infection

**Fig. 2:**
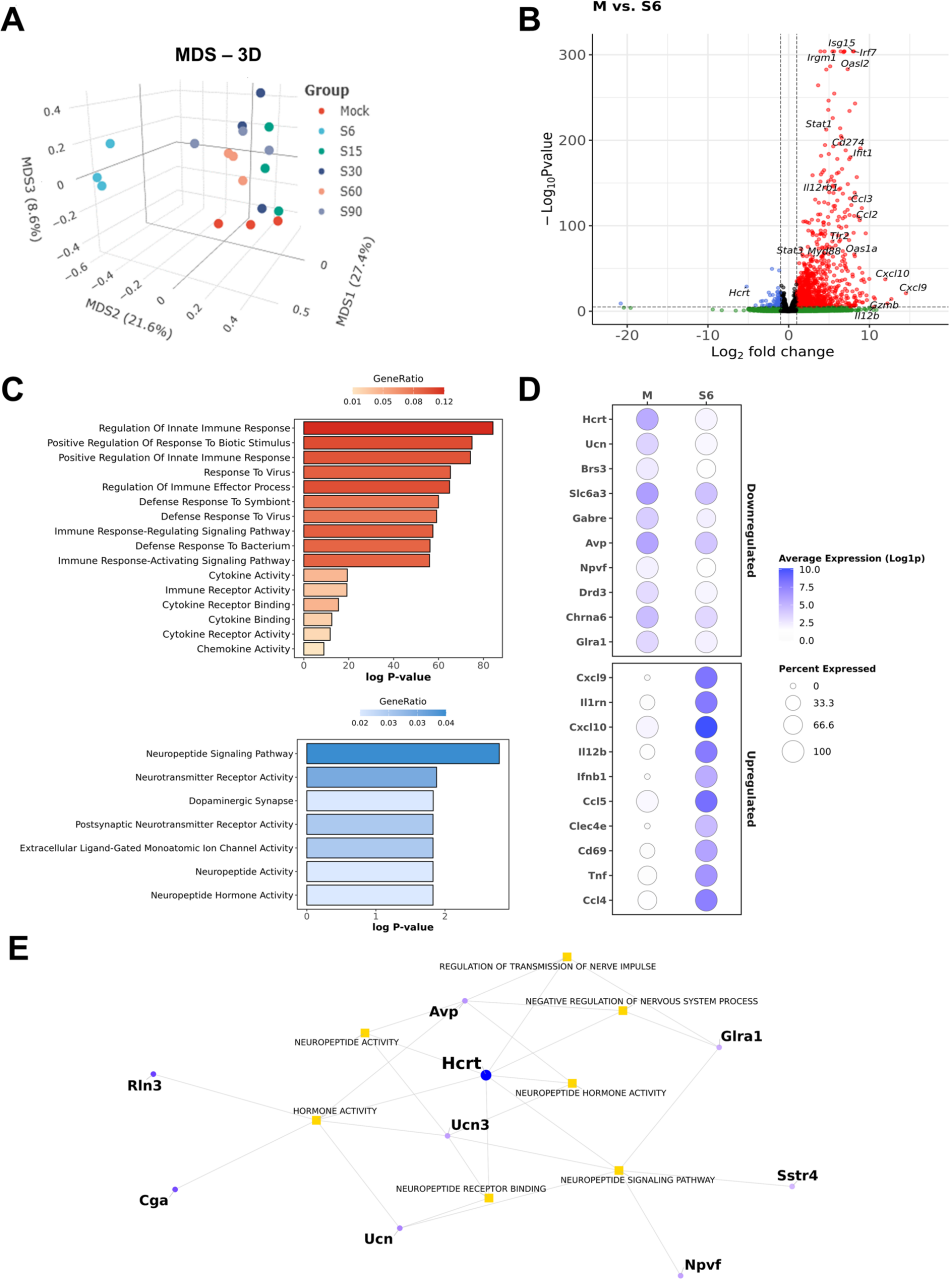
Transcriptomic profiling of SARS-CoV-2-infected K18-hACE2 mouse brains using bulk RNA-seq. **A** Three-dimensional multidimensional scaling (MDS) plot showing segregation of bulk RNA-seq profiles from mock- and SARS-CoV-2-infected brain samples across time points (Mock, 6, 15, 30, 60, and 90 days post-infection (dpi)); percentages on axes indicate variance explained. **B** Volcano plot of differentially expressed genes (DEGs) comparing expression at 6 dpi to the mock controls (M vs. S6). Vertical dashed lines indicate the log_2_ fold change threshold (|log_2_FC| > 1), and the horizontal dashed line indicates the significance threshold (Benjamini–Hochberg adjusted *P* < 0.01; DESeq2). Genes upregulated at 6 dpi relative to the mock treatment (positive log_2_FC) are shown in red, whereas genes downregulated (negative log_2_FC) at this time point are shown in blue. Selected genes, including *Hcrt* are labelled. **C** Gene Ontology (GO) enrichment analysis of the upregulated (top) and downregulated (bottom) DEGs at 6 dpi. Bars represent -log_10_(adjusted *P*) of enrichment with bar colour indicating GeneRatio, defined as the proportion of differentially expressed genes annotated to each GO term. **D** Dot plot showing the expression of selected DEGs with largest absolute log_2_FC across mock and 6 dpi samples, highlighting downregulated neuronal signalling–related genes (top) and upregulated immune-related genes (bottom). Dot size indicates the fraction of samples with detectable expression (non-zero normalised counts) and colour intensity represents the average normalised expression level. **E** Network representation of the downregulated neuropeptide-associated module, identifying *Hcrt* as a central node connected to additional suppressed targets, including *Ucn*, *Avp* and *Npvf*. Bulk RNA-seq was performed on whole- brain tissues (*n* = 3 mice per group per time point). Differential expression was assessed using DESeq2 (Wald test) with |log_2_FC| > 1 and Benjamini–Hochberg adjusted *P* < 0.01

Consistent with the peak whole-brain viral RNA load at 6 dpi (Fig. 1B), we profiled the whole-brain transcriptional responses. Multidimensional scaling (MDS) analysis showed segregation by time point, with 6 dpi separating most prominently from the mock-infected and later samples (Fig. 2A). Differential expression analysis at 6 dpi revealed widespread upregulation of immune and antiviral response genes (Fig. 2B), while Gene Ontology (GO) enrichment of the upregulated transcripts highlighted innate immune categories, including regulation of the innate immune response and defence response to the virus (Fig. 2C, top). Canonical interferon-stimulated and inflammatory genes, including *Isg15*, *Cxcl10*, *Ccl5*, and *Irf7*, were among the most strongly expressed transcripts (Fig. 2B). In parallel, the downregulated transcripts were enriched for neuronal signalling-related terms, including the neuropeptide signalling pathway and neurotransmitter receptor activity (Fig. 2C, bottom), indicating impairment of synaptic transmission. Notably, *Hcrt* (hypocretin/orexin) was the top downregulated transcript in the neuropeptide signalling category (Fig. 2D). Network analysis revealed that *Hcrt* functioned as a central node within the suppressed neuropeptide module, exhibiting functional connectivity with *Ucn*, *Avp* and *Npvf* (Fig. 2E). The concurrent downregulation of Hcrt with stress-regulatory (*Ucn*), neuroendocrine (*Avp*), and sleep-modulating (*Npvf*) factors suggested a coordinated collapse of hypothalamic homeostatic circuits. Pairwise comparisons across time points indicated that the 6 dpi immune transcriptional program was time-restricted, with most genes returning to mock-like levels by 15 dpi, whereas *Hcrt* expression showed a trend toward recovery throughout the post-acute phase (Supplementary Fig. 6).

**Fig. 3:**
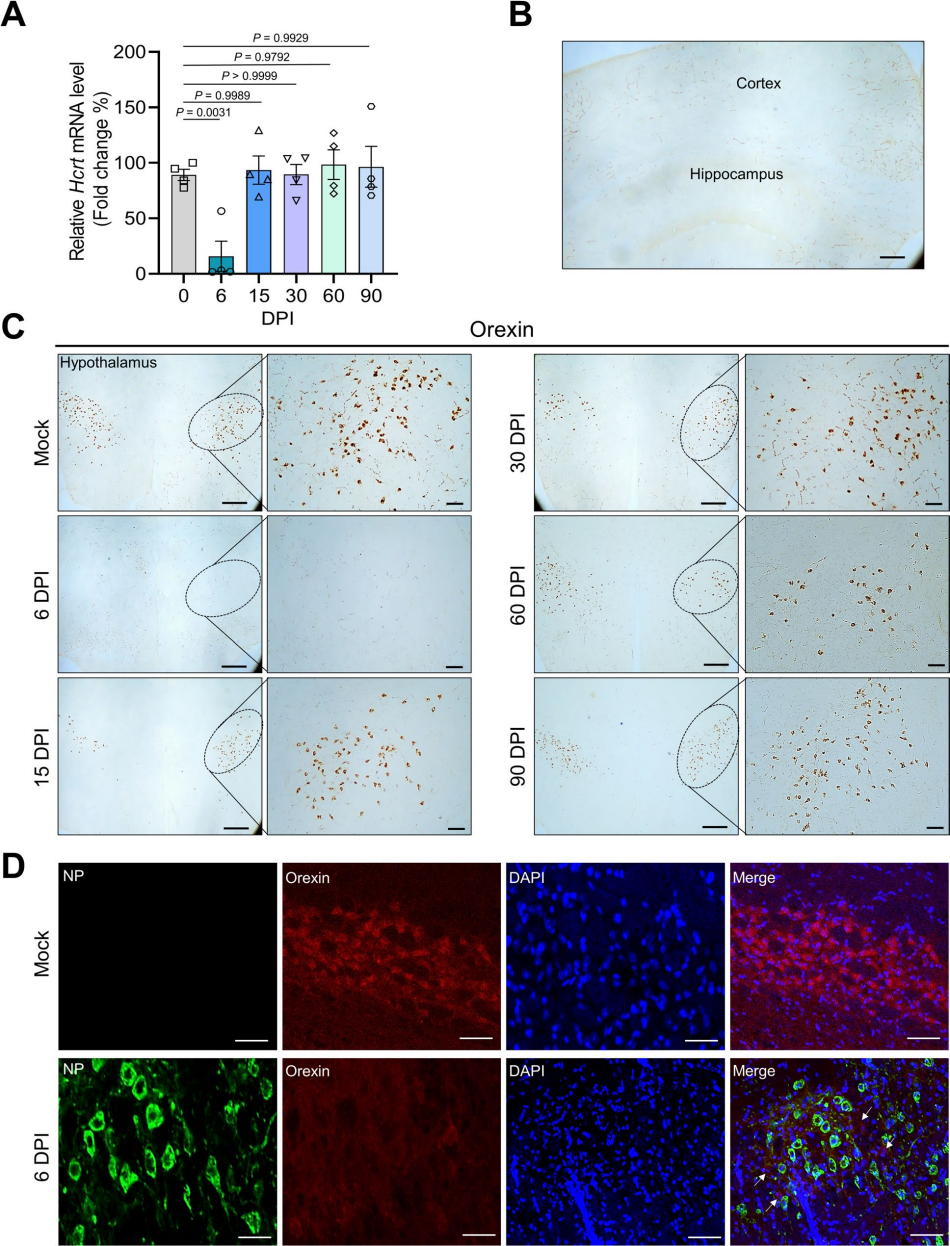
SARS-CoV-2 infection induces transient suppression of hypothalamic hypocretin (orexin) mRNA and protein expression in K18-hACE2 mice. **A** Time-course quantification of Hcrt mRNA levels in whole-brain lysates by RT–qPCR, expressed as fold change relative to mock controls (n = 4 mice per group). **B** Representative low-magnification immunohistochemistry (IHC) image of orexin staining in a mock-infected brain, showing expression restricted to the hypothalamic region. Scale bar, 100 μm. **C** Representative IHC images of orexin immunoreactivity in the lateral hypothalamus at the indicated time points. Dashed ovals highlight the orexin neuron field. High-magnification insets (right) show individual neuronal morphology. Scale bars, 100 μm (low magnification), 30 μm (high magnification). **D** Double immunofluorescence staining for SARS-CoV-2 nucleocapsid (NP; green) and orexin (red) in the hypothalamus in mice infected with SARS-CoV-2 (2 × 104 PFU) at 6 days post-infection (dpi). Nuclei were counterstained with DAPI (blue). White arrows indicate orexin-positive neurons in the NP-negative regions. Scale bar, 25 μm. Representative images in (**B**-**D**) from *n* = 3–5 independent biological replicates are shown. Data represent the mean ± s.e.m. Statistical significance was determined using one-way ANOVA with Dunnett’s multiple comparisons test. The exact *P* values are indicated

Targeted RT–qPCR and histological analyses were performed to validate these signatures. *Hcrt* mRNA abundance was markedly reduced at 6 dpi and was restored to mock levels by 15 dpi (Fig. 3A). Immunohistochemical staining confirmed that orexin immunoreactivity was restricted to the hypothalamus (Fig. 3B) and profoundly diminished at 6 dpi, and re-emerged from 15 dpi onwards (Fig. 3C). Semi-quantitative counting of orexin-positive cells in the lateral hypothalamus supported these histological observations and confirmed a marked reduction at 6 dpi, followed by recovery at later time points (Supplementary Fig. 7). Next, we examined the spatial relationship between the viral antigen and orexin signal using double immunofluorescence staining, which revealed that the orexin signal was minimal within the NP-rich areas, whereas adjacent NP-negative regions retained detectable staining (Fig. 3D, white arrows). Taken together, these data show that acute SARS-CoV-2 infection is associated with the suppression of *Hcrt* transcription and reduced hypothalamic orexin immunoreactivity.

### Brain viral burden associates with downregulation of hypothalamic *Hcrt* expression

**Fig. 4:**
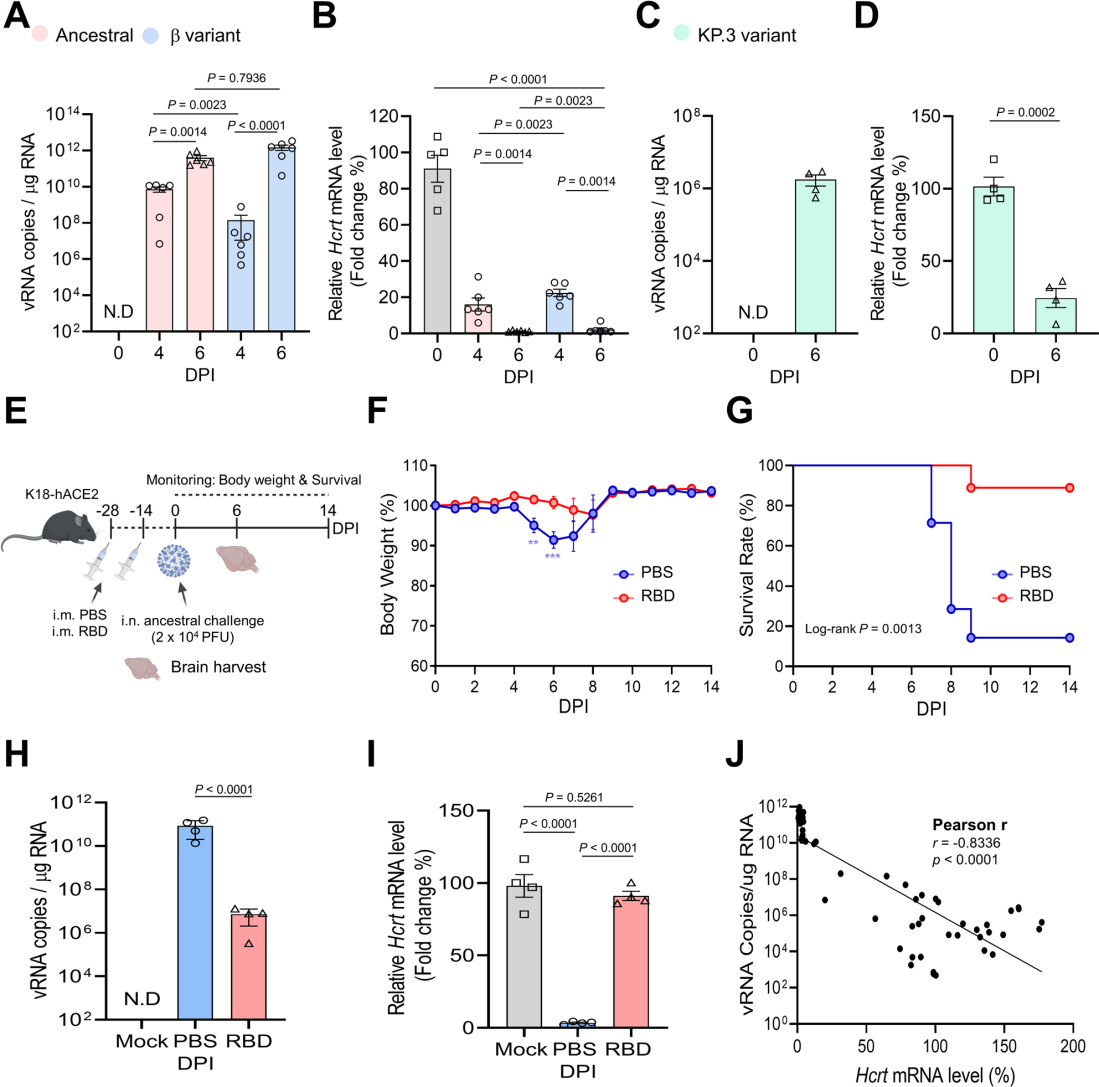
Inverse correlation between SARS-CoV-2 viral burden and transcriptome level of *Hcrt* in K18-hACE2 mice. **A**-**B** Quantification of brain viral RNA load (A) and relative *Hcrt* mRNA levels (B) at 0, 4, and 6 day post-infection (dpi) following infection with ancestral or beta SARS-CoV-2 variants (2 × 103 PFU) (n = 5–6 mice per group). **C**-**D** Quantification of brain viral RNA **C** and *Hcrt* mRNA levels **D** at 6 dpi following infection with the Omicron KP.3 sublineage (n = 4 mice per group). **E** Schematic representation of the vaccination challenge experiment. K18-hACE2 mice were intramuscularly immunised with two doses of RBD subunit vaccine or PBS and challenged with ancestral SARS-CoV-2 (2 × 104 PFU). **F**-**G** Body weight change **F** (n = 11–13 mice per group) and survival rate **G** (*n* = 7–9 mice per group) of vaccinated (RBD) and control (PBS) mice following challenge. For survival analysis, mice were included only if complete follow-up was available through the endpoint, whereas body weight analyses included all animals with longitudinal measurements up to censoring. **H**-**I** Quantification of brain viral RNA load (**H**) and *Hcrt* mRNA levels (**I**) at 6 dpi in vaccinated and control mice (*n* = 4 mice per group). **J** Correlation analysis between brain viral RNA load and *Hcrt* mRNA levels using pooled data from all experimental cohorts (*n* = 56 paired samples). Pearson’s correlation coefficients (r) and P values are indicated. Data represent the mean ± s.e.m. Statistical significance was determined using one-way ANOVA with Dunnett’s multiple comparisons test (**A**), (**B**), (**I**), two-tailed unpaired Student’s t-test (**C**), (**D**), (**H**), two-way ANOVA with repeated measures followed by Sidak’s multiple comparisons test (**F**), or log-rank test (**G**). N.D., not detected

To determine whether *Hcrt* downregulation is associated with the viral burden in the brain, we quantified the viral RNA and *Hcrt* mRNA across SARS-CoV-2 variants. Upon reanalysing brain tissue from our cohort infected with ancestral (Wuhan-Hu-1) and Beta (B.1.351) SARS-CoV-2 variants [28], we observed that brain viral RNA increased between 4 and 6 dpi (Fig. 4A), coincident with a progressive decline in *Hcrt* mRNA. In both groups, *Hcrt* expression declined to ~ 15–20% of the mock expression at 4 dpi and to below 2% by 6 dpi (Fig. 4B). Infection with the Omicron sublineage KP.3 likewise resulted in a robust brain viral burden at 6 dpi, with *Hcrt* expression at approximately 25% of the mock levels, indicating that *Hcrt* mRNA suppression is conserved across variants (Fig. 4C and D). Next, we investigated whether preventing viral replication through vaccination could rescue *Hcrt* expression. A recombinant RBD subunit protein formulated with alum was administered intramuscularly twice at 2-week intervals, followed by a SARS-CoV-2 challenge (Fig. 4E). Vaccinated mice maintained stable body weight and showed markedly improved survival rates (88%) than the PBS controls (14%) (Fig. 4F and G). At 6 dpi, vaccination reduced the viral RNA levels in the brain by ~ 10^4^-fold (Fig. 4H). Notably, *Hcrt* mRNA levels remained near the mock levels (~ 90%) in vaccinated mice, rather than decreasing to 3.5%, as in PBS controls (Fig. 4I). Across pooled datasets, brain viral RNA exhibited a strong inverse association with *Hcrt* mRNA abundance (Pearson *r* = − 0.8336; Fig. 4J). Together, these data indicate that suppression of hypothalamic *Hcrt* mRNA inversely associates with brain viral RNA and that vaccine-mediated reductions in brain viral RNA coincide with preservation of *Hcrt* expression.

### MA10 SARS-CoV-2, but not influenza A virus, recapitulates cortical NeuN loss and hypothalamic orexin depletion in wild-type mice

**Fig. 5:**
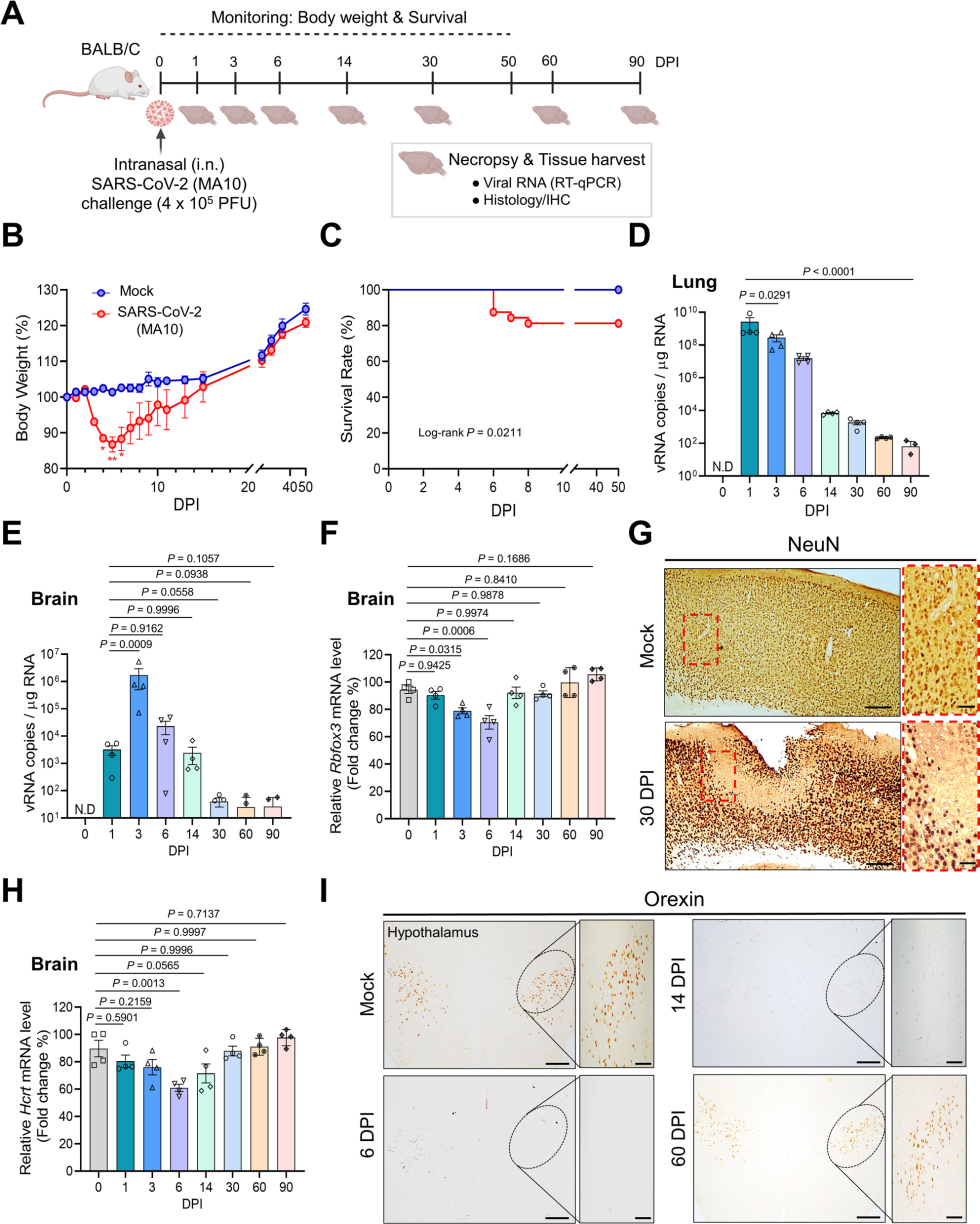
MA10 SARS-CoV-2 infection causes persistent cortical NeuN reduction and hypothalamic orexin suppression in wild-type mice. **A** Schematic of the experimental design. BALB/c mice were intranasally challenged with mouse-adapted SARS-CoV-2 (MA10; 4 × 105 PFU) and monitored for up to 90 days post-infection (dpi); tissues were collected for viral RNA quantification and histopathology at the indicated time points. (**B**-**C**) Body weight change (**B**) (mock *n* = 5, MA10 *n* = 12 mice, per group) and survival (C) (mock *n* = 26, MA10 *n* = 32 mice per group) of MA10-infected mice compared with mock controls. **D**-**F** Time-course quantification of lung viral RNA (**D**), brain viral RNA (**E**), and Rbfox3 (NeuN) mRNA levels (**F**) at the indicated time points (*n* = 4 mice per group per time point). **G** Representative NeuN immunohistochemistry (IHC) results in the cerebral cortex of mock- and MA10-infected mice at 30 days post-infection (dpi). Red dashed boxes indicate regions with reduced NeuN immunoreactivity. Scale bars, 100 μm. Representative images from n = 3–4 independent biological replicates are shown. **H** Temporal profile of Hcrt mRNA expression in MA10-infected brains (*n* = 4 mice per group). **I** Representative orexin IHC in the lateral hypothalamus of mock- and MA10-infected BALB/c mice at 6, 14, and 60 dpi. Dashed ovals highlight the orexin neuron field. Higher-magnification views show orexin-positive neurons. Scale bars, as indicated. The dashed line indicates the limit of detection. Data are the mean ± s.e.m. Statistical significance was determined using two-way ANOVA with repeated measures followed by Sidak’s multiple-comparison test (**B**), log-rank (Mantel–Cox) test (**C**) and one-way ANOVA with Dunnett’s multiple-comparisons test (**D**, **E**, **F**, **H**). The exact *P* values are indicated or denoted as *P < 0.05, **P < 0.01. N.D., not detected

To validate physiological conditions, we extended our analysis to mouse-adapted SARS-CoV-2 (MA10) in wild-type BALB/c mice (Fig. 5A), which recapitulated the key clinical and pathological features of COVID-19 [29, 30]. The infected mice exhibited transient weight loss (~ 88% at 5 dpi), followed by rapid recovery (Fig. 5B). Consistent with previous reports [29], infection with 4 × 10^5^ PFU led to a high survival rate (approximately 80%), enabling long-term monitoring (Fig. 5C). Next, we quantified the viral burden to assess neuroinvasion dynamics. The viral RNA levels in the lung peaked at 1 dpi and declined thereafter (Fig. 5D). Viral RNA levels in the brain peaked at 3 dpi and then gradually decreased (Fig. 5E). RT-qPCR revealed a ~ 30% reduction in cortical *Rbfox3* (NeuN) mRNA expression at 6 dpi (Fig. 5F). Beyond this transcriptional deficit, immunohistochemical analysis revealed persistent NeuN-depleted regions in the cerebral cortex up to 90 dpi, whereas the olfactory bulb and hippocampus remained unaffected (Supplementary Fig. 8). Discrete, patchy cortical regions of reduced NeuN immunoreactivity emerged stochastically, with high-magnification images confirming marked NeuN loss despite preserved neuronal density but frequent shrunken morphology on Nissl staining (Fig. 5G and Supplementary Fig. 8A and B). The representative cortical NeuN-depleted regions shown in Fig. 5G were further assessed by semi-quantitative area analysis (Supplementary Fig. 9A). Similarly, in the MA10 model, the NeuN-low phenotype was not confined to a reproducible cortical subregion and did not show a consistent laminar distribution across animals. Targeted RT-qPCR investigating hypothalamic integrity revealed pronounced depletion of *Hcrt* transcripts (~ 40% reduction at 6 dpi), which persisted at 14 dpi (Fig. 5H). Immunohistochemistry confirmed that the reduced orexin expression was restricted to the lateral hypothalamus (Fig. 5I). Semi-quantitative counting of orexin-positive cells in the lateral hypothalamus likewise confirmed the reduction observed in representative hypothalamic sections following MA10 infection (Supplementary Fig. 9B). These findings demonstrate that MA10 infection in wild-type mice reproduces the distinctive cortical and hypothalamic neuropathology observed in K18-hACE2 mice.

**Fig. 6:**
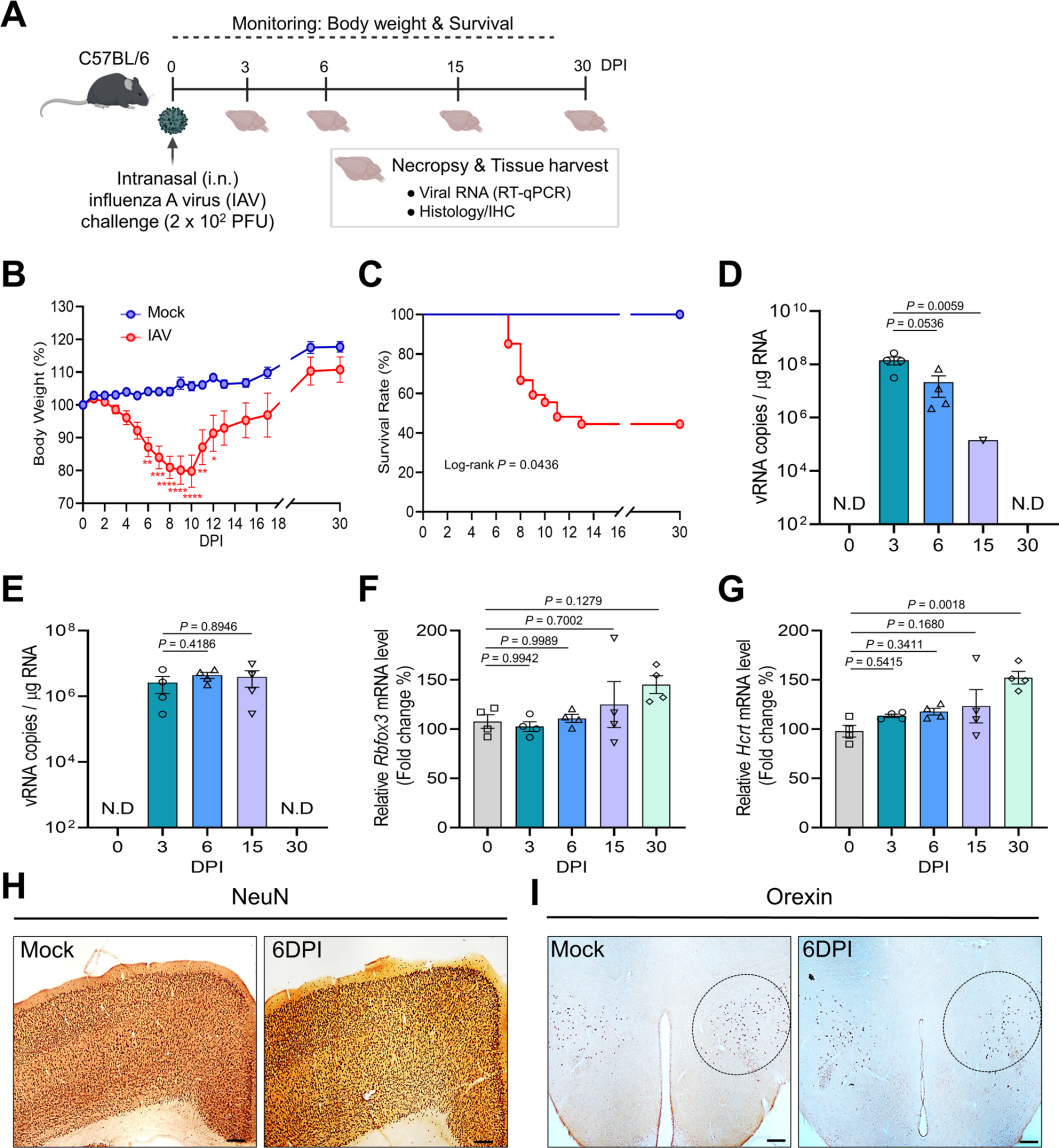
Cortical NeuN expression and hypothalamic orexin levels are preserved in influenza A virus-infected mice. **A** Schematic of the experimental design. C57BL/6 mice were intranasally challenged with the influenza A virus (IAV; PR8 strain, 2 × 102 PFU). **B**-**C** Body weight change (**B**) (Mock, *n* = 5, IAV, *n* = 15) and survival analysis (**C**) (Mock *n* = 5, IAV *n* = 27) of IAV-infected mice compared with Mock controls. **D**-**E** Time-course quantification of lung (**D**) and brain **E** viral RNA, and (**F**-**G**) Rbfox3 (**F**), and Hcrt mRNA (**G**) levels in the brain at the indicated time points (*n* = 4 mice per group per time point). **H**-**I** Representative IHC staining for NeuN in the cortex (**H**) and for orexin in the hypothalamus (**I**) of Mock- and IAV-infected mice at 6 dpi. Scale bars, 100 μm. Representative images from *n* = 3–4 independent biological replicates are shown. Data represent the mean ± s.e.m. Statistical significance was determined using two-way ANOVA with repeated measures followed by Sidak’s multiple comparison test (**B**), log-rank (Mantel–Cox) test (**C**) or one-way ANOVA with Dunnett’s multiple comparisons test (**D**-**G**). The exact *P* values are indicated by asterisks as follows: *P < 0.05, **P < 0.01, ***P < 0.001, and ****P < 0.0001. N.D., not detected

To determine whether these lesions are specific to SARS-CoV-2 or represent a generalised consequence of severe respiratory viral infection, we compared these outcomes with those in C57BL/6 mice infected intranasally with influenza A virus (IAV; H1N1 strain PR8) (Fig. 6A). IAV infection led to progressive morbidity, with body weight decreasing to ~ 80% by 9 dpi and survival reduced to ~ 45% (Fig. 6B and C). Despite robust pulmonary replication (Fig. 6D) and brain viral RNA levels equivalent to those with MA10 infection (Fig. 6E), transcriptional analysis revealed no significant changes in *Rbfox3* mRNA (Fig. 6F) and NeuN immunoreactivity (Fig. 6H). Furthermore, *Hcrt* mRNA (Fig. 6G) and orexin expression levels remained unaltered (Fig. 6I).

Together, these data reveal that, although MA10 SARS-CoV-2 infection induces prominent cortical neuronal dysfunction and hypocretin depletion, IAV infection does not, despite comparable levels of brain-associated viral RNA detected under the tested conditions. These findings suggest that, in the models tested here, suppression of the hypocretin system and associated cortical neuronal damage are more closely linked to SARS-CoV-2 infection than to the mere presence of brain-associated viral RNA.

### Exogenous orexin-A/B increases NeuN abundance under SARS-CoV-2 infection-related experimental conditions

**Fig. 7:**
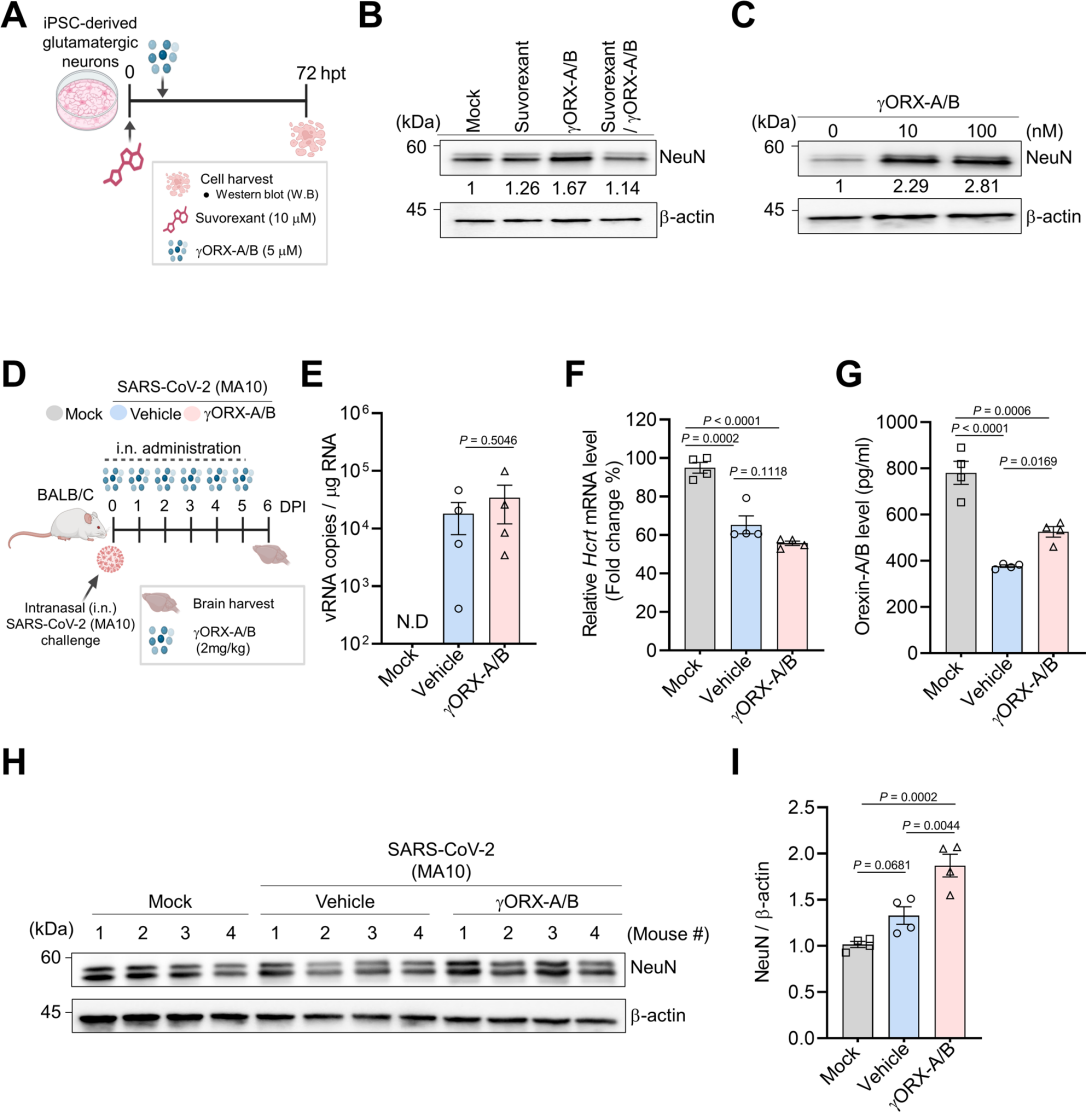
Exogenous orexin signalling promotes NeuN expression in vitro and in vivo during SARS-CoV-2 infection. **A** Schematic illustration of the in vitro experimental design used to assess the specificity of orexin signalling. Human iPSC-derived glutamatergic neurons were pre-treated with the dual orexin receptor antagonist suvorexant (10 µM) or vehicle for 2 h, followed by treatment with recombinant orexin-A/B peptides (γORX-A/B; 5 µM each) at the indicated concentrations; the cells were then harvested at 72 h post-treatment (hpt) for immunoblotting. **B** Western blot analysis showing the effect of suvorexant pretreatment on γORX-A/B-mediated NeuN induction. Representative blots (top) and quantification of NeuN protein levels normalized to β-actin (bottom) are shown. The fold change relative to the mock control is indicated below the blots. **C** Dose-dependent effects of orexin on NeuN expression. Neurons were treated with increasing concentrations of γORX-A/B (0, 10, and 100 nM) and analysed by western blotting at 72 h post-treatment (hpt). **D** Schematic representation of the in vivo orexin treatment experiments. BALB/c mice were intranasally infected with MA10 SARS-CoV-2 (4 × 10^4^ PFU) and administered γORX-A/B (2 mg kg^-1^) or vehicle daily from 0 to 5 days post-infection (dpi). Brain tissues were collected at 6 dpi. **E**-**F** Quantification of viral burden (**E**) and Hcrt mRNA levels (**F**) in whole-brain lysates at 6 dpi using RT–qPCR. **G** ELISA quantification of the orexin-A/B peptide concentrations in brain homogenates. **H** Representative Western blots of NeuN expression in whole-brain lysates from mock, vehicle-treated and γORX-A/B-treated mice at 6 dpi. **I** Densitometric quantification of NeuN protein levels normalised to β-actin. Data in (**E**-**G**) and (**I**) represent mean ± s.e.m. (n = 4 mice per group). Statistical significance was determined using one-way ANOVA with Dunnett’s multiple comparison test (**F**, **G**, **I**) or a two-tailed unpaired Student’s t-test (**E**). The exact *P* values are indicated. N.D., not detected

We next asked whether exogenous orexin-A/B supplementation could influence NeuN-associated readouts under our experimental conditions (Fig. 7A). Human iPSC-derived glutamatergic neurons, which lack major CNS inflammatory effector cell types such as microglia and astrocytes, and do not exhibit endogenous orexin signalling under standard culture conditions [31], supported productive SARS-CoV-2 infection, but NeuN expression was not reduced under the tested in vitro conditions at 72 hpt (Supplementary Fig. 10A–C). This result suggested that direct viral infection alone was not sufficient to recapitulate the cortical NeuN attenuation observed in vivo, and prompted us to examine whether exogenous orexin-A/B could modulate NeuN abundance in this system. Treatment with recombinant orexin-A/B increased NeuN abundance by ~ 1.7-fold compared with mock controls, whereas pretreatment with the dual orexin receptor antagonist suvorexant reduced this effect (Fig. 7B). Dose-response analysis showed that 10 nM and 100 nM γORX-A/B were associated with increased NeuN levels of ~ 2.3-fold and ~ 2.8-fold, respectively (Fig. 7C). To examine the effects of exogenous orexin-A/B in vivo, BALB/c mice were infected with MA10 SARS-CoV-2 and treated intranasally with γORX-A/B daily from 0 to 5 dpi (Fig. 7D). For this treatment experiment, a lower MA10 inoculation dose (4 × 10^4^ PFU) than used in Fig. 5 was intentionally selected to reduce lethality and enable assessment of ORX-A/B effects under a less severe infection condition. At 6 dpi, the brain viral loads were comparable between the vehicle- and γORX-treated groups (Fig. 7E), indicating that orexin administration does not alter viral replication. *Hcrt* mRNA levels were reduced by approximately 40% in both groups (Fig. 7F), consistent with a sustained endogenous orexin deficit, whereas total ORX-A/B levels were significantly higher in γORX-treated brains (Fig. 7G), indicating efficient peptide delivery. Western blot analysis of whole-brain lysates showed that total NeuN levels in vehicle-treated infected mice were comparable to those in mock-infected controls, whereas γORX-A/B treatment was associated with an increase in NeuN abundance (Fig. 7H). Quantification showed that NeuN protein abundance was approximately 1.8-fold higher in γORX-A/B-treated mice than in mock controls (Fig. 7I). Because this analysis was performed using whole-brain lysates, localized cortical NeuN attenuation identified histologically may not have been fully captured at the level of total brain protein.

Collectively, these data show that exogenous orexin-A/B increased NeuN abundance under infected conditions in vivo, without altering viral load. These findings support an association between orexinergic signalling and NeuN-associated neuronal state during SARS-CoV-2 infection, while not establishing formal biochemical rescue of a whole-brain NeuN deficit or a direct causal mechanism.

## Discussion

SARS-CoV-2 is increasingly recognised as a cause of persistent neurological sequelae, yet the mechanisms linking acute infection with long-term circuit dysfunction remain unclear. We identified two convergent in vivo signatures of SARS-CoV-2 neuropathology: focal, heterogeneous loss of cortical NeuN immunoreactivity persisting beyond the acute phase, and selective suppression of hypothalamic hypocretin/orexin (*Hcrt*) expression during early infection. Using comparative viral models and pharmacological interventions, we revealed that these processes are connected through orexinergic circuit disruption, suggesting a pathway that bridges acute infections to lasting neuronal vulnerability. This mechanism provides a potential cellular basis for Long COVID symptoms [32], including sleep–wake cycle instability, fatigue, and cognitive dysfunction.

Neuroinflammation is a hallmark of respiratory viral infection and can profoundly influence neuronal function; however, whether inflammation alone explains persistent, spatially restricted neuronal dysfunction remains uncertain. In our analysis, inflammatory mediators such as Cxcl10 and Ccl2 surged during the acute phase (Fig. 1C), consistent with reports of blood–brain barrier disruption and cytokine induction [33, 34]—, but subsided thereafter, whereas the downregulation of focal cortical NeuN persisted. This temporal dissociation indicates that early cytokine elevation cannot fully account for the enduring and anatomically focal NeuN downregulation (Supplementary Fig. 4). Comparative viral evidence reinforces the “inflammation-is-not-enough” model: peripheral H1N1 influenza infection triggers robust neuroimmune activation, but does not produce the observed shrinkage or NeuN loss in neurons [13, 14]. Thus, the mammalian brain can withstand substantial inflammatory stress without neurodegeneration, implying that persistent cortical pathology requires mechanisms beyond general neuroinflammatory signalling. This is consistent with recent CSF profiling in Myalgic Encephalomyelitis/Chronic Fatigue Syndrome (ME/CFS), which suggests that distinct central immune signatures, rather than generalised inflammation alone, drive specific neurological phenotypes [35–38].

We tracked the mature neuronal marker NeuN (*Rbfox3*) and observed sustained, spatially “patchy” reduction in cortical NeuN immunoreactivity, appearing as stochastic multifocal clusters. Nissl staining indicated that neurons within NeuN-attenuated foci were not absent but often appeared shrunken or atrophic. Importantly, loss of NeuN antigenicity does not necessarily indicate neuronal death but often reflects metabolic stress or transcriptional suppression in structurally preserved neurons, as shown in ischaemic models [39–41]. Nonetheless, persistent NeuN attenuation indicates a chronically compromised neuronal state that predisposes patients to delayed neuronal degeneration or dysfunction [42]. Thus, following SARS-CoV-2 infection, neurons survive but adopt a shrunken, transcriptionally altered state, which may contribute to the circuit instability and cognitive ‘brain fog’ associated with Long COVID. The apparent discrepancy between whole-brain Rbfox3 mRNA recovery and persistent focal cortical NeuN attenuation should be interpreted cautiously. Because the transcript analysis was performed on whole-brain homogenates whereas the histological changes were spatially restricted within the cortex, localized NeuN-low regions may have been diluted at the whole-brain level. This divergence may also reflect compensatory changes in unaffected regions or altered NeuN antigenicity, as discussed above.

The next question was whether these neuropathological features depend on direct viral access to the CNS. Our data identified SARS-CoV-2 as a neurotropic virus capable of entering the CNS and exhibiting tropism for hypothalamic circuits. High-resolution imaging at 6 dpi revealed an inverse spatial relationship between viral burden and orexin expression: orexin immunoreactivity was virtually absent in regions dense with viral NP signals, whereas adjacent NP-negative zones retained detectable staining (Fig. 3D, white arrows). This spatial exclusivity suggests that orexin loss arises from local viral infection rather than systemic inflammation. Human autopsies and animal studies likewise confirm SARS-CoV-2 neuroinvasion [10, 11]. Similar patterns are found in neuroadapted influenza strains; for instance, neurotropic H1N1 invades via the olfactory route to destroy orexin neurons and induce narcolepsy-like sleep disruption [43] and H7N7 infection drives microglial activation and synaptic loss [44]. In contrast, our PR8 (H1N1) model—, a well-characterised non-neurotropic strain that induces severe systemic inflammation but lacks intrinsic neuronal replicative capacity—, showed comparable levels of brain-associated viral RNA under the tested conditions, but did not elicit hypothalamic orexin dysfunction or cortical NeuN attenuation. Detection of PR8 viral RNA in whole-brain samples should be interpreted cautiously, as this does not by itself establish productive CNS infection. Prior studies have shown that non-neurotropic PR8 can be detected in olfactory bulb or brain-associated compartments under some intranasal infection conditions without implying widespread productive neuronal infection [45, 46]. This dissociation suggests that systemic inflammatory severity and the mere presence of viral RNA in the brain are insufficient, by themselves, to account for the observed phenotype. A limitation of the MA10-versus-PR8 comparison is that these infections were performed in different mouse strains (BALB/c versus C57BL/6), and strain-dependent differences in immune and neuroinflammatory responses may influence the observed phenotypes. However, because SARS-CoV-2-associated hypothalamic orexin suppression and cortical NeuN attenuation were also observed in the C57BL/6-based K18-hACE2 model [25, 26], the current findings are not readily explained by strain background alone, although definitive virus specificity will require future same-strain comparative studies.

We therefore propose a working model in which selective hypothalamic orexin suppression is associated with cortical NeuN attenuation during SARS-CoV-2 infection. Our transcriptomic analysis revealed synchronised suppression of *Hcrt* along with *Ucn*, *Avp* and *Npvf*, consistent with a coordinated perturbation of hypothalamic homeostatic programs. Given that CRF/urocortin-family signalling and vasopressin directly modulate orexin neuronal activity and orexin-dependent control of stress-associated arousal and wakefulness [47, 48], and that Neuropeptide VF (Npvf) exhibits evolutionarily conserved links to orexin cell states and behavioural-state regulation [49, 50], their concurrent downregulation implies a systemic failure of the arousal network. Orexin neurons serve as integrative hubs that regulate sleep—wake cycle stability, arousal, and neuroendocrine balance, while maintaining neuroprotection and immune homeostasis [51]. Orexin-A promotes neuronal survival under oxidative stress [20], supports hippocampal neurogenesis [52] and limits neuroinflammation via NF-κB inhibition [21]. This central role aligns with the emerging evidence of orexinergic disruption in COVID-19, including autoantibodies against orexin receptors [53]. To separate the direct viral effects from circuit-level mechanisms, we used human iPSC-derived cortical neurons, which supported viral replication but maintained NeuN expression without orexin signalling (Supplementary Fig. 10), indicating that direct infection alone was insufficient for NeuN loss. Conversely, exogenous orexin upregulated the NeuN levels in a receptor-dependent manner without altering the viral burden (Fig. 7). However, because the in vivo biochemical analysis was performed using whole-brain lysates, the focal cortical NeuN-low phenotype may not have been fully captured at the level of total brain protein. Accordingly, the increased NeuN abundance observed after γORX-A/B treatment should be interpreted cautiously and does not by itself establish definitive histological rescue of the focal cortical phenotype. Overall, these data suggest that virus-mediated disruption of the orexin system offers a plausible basis for the fatigue, arousal deficits, and cognitive dysfunction characteristic of Long COVID [32]. Previous studies have reported OX1R expression in cortical regions, as well as expression of both OX1R and OX2R in cultured cortical neurons, supporting the anatomical plausibility that cortical neurons can respond to orexin signalling [54]. However, receptor localization was not directly examined in the present infected mouse model, and future studies will be needed to define OX1R/OX2R distribution in NeuN-depleted versus intact cortical regions. It also remains unknown whether SARS-CoV-2 proteins directly interact with orexin or orexin receptors.

The K18-hACE2 transgenic mouse is a standard model for severe COVID-19 and has been used to assess vaccine efficacy and respiratory disease pathology [34, 55]. Beyond lung injury, it recapitulates extrapulmonary symptoms, including retinal inflammation and ocular discharge, which mirror conjunctivitis in patients [56, 57]. These manifestations align with known neuronal dissemination pathways connecting the respiratory, neural, and ocular tissues, supporting their use in studying SARS-CoV-2 neuroinvasion [10, 57]. In this model, infection reduced hypothalamic orexin and cortical NeuN expression, a phenotype conserved across variants including Beta and Omicron KP.3 (Fig. 4A-D), indicating that orexin dysfunction is an intrinsic feature of SARS-CoV-2 neuropathology. To confirm its physiological relevance, we analysed MA10 SARS-CoV-2 infection in wild-type BALB/C mice [58], which reproduced the same deficits. Unlike K18-hACE2 mice, which showed orexin recovery by 15 dpi (Fig. 3A), MA10-infected mice showed prolonged orexin suppression through 14 dpi (Fig. 5I) and persistent cortical NeuN loss up to 90 dpi (Supplementary Fig. 8A). This sustained pathology in wild-type mice demonstrates that SARS-CoV-2-induced neuropathology is a robust and intrinsic outcome, and not an artifact of transgenic ACE2 expression.

This study has several limitations that should be considered in interpreting our findings and in designing for future studies. First, the K18-hACE2 mouse model artificially overexpresses human ACE2, which facilitates widespread viral neuroinvasion and leads to fulminant CNS pathology that likely exceeds the typical milder or more restricted neurological involvement seen in most human SARS-CoV-2 infections. Furthermore, because our long-term assessments (up to 90 dpi) relied on the surviving fraction of mice after low-dose challenge, these observations may be influenced by survivorship bias and may reflect a specific immunological subset rather than the general infected population. Although we partially mitigated these translational gaps by demonstrating similar neuropathology in wild-type BALB/c mice infected with the mouse-adapted MA10 strain, our findings should be interpreted as a discovery-oriented mechanistic framework for post-viral neurological sequelae rather than a direct replication of human Long COVID. Second, NeuN attenuation alone does not indicate neuronal death. In the present study, limited TUNEL-positive signal was observed in the cortex at 6 dpi (Supplementary Fig. 11); however, its correspondence with focal NeuN-low regions was insufficient to conclude that this phenotype primarily reflects widespread apoptotic neuronal death. Thus, the fate of these neurons remains unresolved. Additional analyses using complementary markers such as Fluoro-Jade, cleaved caspase-3, and synaptic injury markers across extended time points will be required to determine whether the NeuN-low phenotype reflects reversible dysfunction, delayed degeneration, or overt cell death [39]. Third, whether orexin deficiency is causal or correlative remains unresolved. Although exogenous orexin-A/B increased NeuN abundance under our experimental conditions, these data do not establish that endogenous orexin loss directly drives cortical NeuN attenuation. In addition, the in vivo biochemical analysis was performed on whole-brain lysates, which may not fully capture focal cortical NeuN-low regions identified histologically. Targeted loss-of-function perturbations of orexin neurons or receptors, together with region-resolved analyses and longitudinal behavioural profiling, will be needed to establish causality [59]. Furthermore, unlike hypothalamic orexin neurons, the iPSC-derived glutamatergic neurons used in this study do not secrete orexin; therefore, extending the mechanistic analyses to orexinergic neuronal models, including co-expressed markers such as dynorphin, will clarify whether orexin-producing cells themselves are directly vulnerable to infection. In addition, the current study did not include behavioural assessments relevant to sleep/wake regulation, fatigue, or cognition. Because the available ABSL-3 facility was not configured for longitudinal behavioural monitoring, future studies, ideally in collaboration with groups equipped for behavioural phenotyping, will be required to directly link the observed molecular and histological changes to functional neurological outcomes relevant to Long COVID. Finally, only male mice were used in the present study. This is an important limitation because long COVID risk appears to be higher in females in human cohorts, and both neuroimmune responses and the orexin/hypocretin system show sex-dependent features [60]. Although male mice may exhibit greater susceptibility in some acute SARS-CoV-2 infection models [61], future studies including female mice will be required to determine whether the orexin-associated neuropathological phenotype described here is sex dependent.

Collectively, our findings identify hypocretin/orexin suppression as a selective feature of SARS-CoV-2 brain pathology that coincides with persistent, spatially heterogeneous cortical NeuN attenuation. Exogenous orexin-A/B supplementation increased NeuN abundance without altering viral load, supporting a possible link between orexinergic dysfunction and neuronal vulnerability during SARS-CoV-2 infection. However, further mechanistic studies will be required to determine whether endogenous orexin loss directly contributes to the arousal and cognitive impairments associated with Long COVID.

## Methods

### Ethics statement and biosafety

All animal procedures were approved by the Institutional Animal Care and Use Committee (IACUC) of the Korea Research Institute of Chemical Technology (KRICT) and conducted in accordance with the institutional guidelines and relevant national regulations (Protocol ID 8 A-M6; IACUC IDs 2023–8 A-07-01, 2023–8 A-08-02, 2025–8 A-04-03 and 2025–8 A-08-02). Experiments involving infectious SARS-CoV-2 were performed in biosafety level 3 (BSL-3) facilities at KRICT, and experiments involving influenza A virus (IAV; H1N1) were performed in biosafety level 2 (BSL-2) laboratories. All studies were performed in compliance with the relevant national and institutional biosafety regulations. As this work did not involve a clinical trial, no clinical trial registration number is applicable.

### Animals and in vivo procedures

Male B6.Cg-Tg(K18-hACE2)2Prlmn/J mice were purchased from The Jackson Laboratory (Bar Harbor, ME, USA). Male BALB/cAnNCrlOri and C57BL/6NCrlOri mice were obtained from Orient Bio Inc. (Seongnam, Republic of Korea). Animals were maintained in a BSL-2 animal facility in individually ventilated cages at the Korea Research Institute of Chemical Technology (KRICT), with controlled temperature (22 ± 2 °C), humidity (50 ± 10%), and a 12-h light/dark cycle. The experimental groups comprised age-matched mice (8–9 weeks old).

All viral inoculations [SARS-CoV-2 (50 PFU), MA10 SARS-CoV-2 (4 × 10⁵ PFU), and influenza A virus (2 × 10² PFU)] were administered intranasally (IN) at a volume of 20 µL per mouse, under isoflurane anaesthesia in BSL-3 or BSL-2 animal facilities, as appropriate, and all efforts were made to minimise animal suffering. IN inoculations were performed as previously described [57]. Mock-infected mice were administered an equal volume of PBS. Post-infection, body weight and survival were monitored daily. At designated time points following infection (as indicated in the respective figure legends), mice were deeply anaesthetized with isoflurane and perfused transcardially with PBS, followed by 4% paraformaldehyde (PFA) for histological assessment, or fresh tissues were harvested and flash-frozen for downstream molecular and biochemical analyses.

To evaluate vaccine efficacy, K18-hACE2 mice were immunised intramuscularly (IM) with 10 µg of recombinant SARS-CoV-2 Spike RBD protein formulated using aluminium hydroxide gel (Alum) (Alhydrogel 2%, InvivoGen, San Diego, CA, USA) as an adjuvant in a 1:1 ratio, at a final volume of 100µL per mouse or with PBS/Alum (Mock control). Booster immunisations with the same dose and formulation were administered after two weeks. Two weeks after the final vaccination, mice were challenged intranasally with 2 × 10^4^ PFU of SARS-CoV-2 in a 20 µL volume (100MLD_50_). The mice were then monitored daily for 14 dpi. On day 6 post-infection, brain tissues were collected for downstream molecular and biochemical analyses as described above.

To evaluate the therapeutic effects of orexin in vivo, BALB/c mice were intranasally infected with MA10 SARS-CoV-2 (4 × 10^4^ PFU). Recombinant orexins A and B (2 mg/kg each; MCE, HY-106224B, HY-P1349) or vehicle (PBS) were administered intranasally daily from 0 to 5 dpi. Mice were euthanised at 6 dpi and whole brains were harvested for viral load quantification and western blot analysis.

### Cells and viruses

The ancestral SARS-CoV-2 Korean strain (GISAID: EPI_ISL_407193; NCCP: 43326) and the Omicron KP.3 variant (NCCP: 43496) were obtained from the Korea Disease Control and Prevention Agency (KDCA). The mouse-adapted SARS-CoV-2 MA10 strain was obtained from BEI Resources (NIAID, NIH, USA). All viruses were propagated in Vero cells (CCL-81; ATCC) grown in Dulbecco’s Modified Eagle’s Medium (DMEM) supplemented with 2% foetal bovine serum (FBS) as previously described [58, 62]. The influenza A virus H1N1 (PR8 strain) was kindly provided by Prof. Heung Kyu Lee (KAIST). Viral titres were determined using a plaque assay on Vero cells.

### Differentiation of human iPSC-derived glutamatergic neurons

Glutamatergic neurons were generated from human iPSCs as previously described [31] with minor modifications. Human iPSCs (ATCC-DYR0100; ACS-1011) were cultured to ~ 70% confluence in mTeSR1 medium supplemented with Y-27,632 (10 µM; Millipore Sigma). Cells were dissociated with 0.5 mM EDTA and seeded (3.5 × 10⁵ cells/well) onto Matrigel-coated 6-well plates in chemically defined medium (CDJ; Neurobasal/DMEM-F12 [1:1] supplemented with B27, N2, and penicillin-streptomycin). On the following day, cells were transduced with lentiviral vectors encoding hNGN2 (Addgene 62223) and rtTA (Addgene 20342) at a multiplicity of infection (MOI) of 5 via spin-infection (1000 × g, 1 h). At 24 h post-transduction, medium was replaced with CDM containing doxycycline (2.5 µg/ml) and puromycin (1 µg/ml) for inducing NGN2 expression and selection, respectively. After 48 h of selection, cells were re-seeded on Matrigel-coated plates and maintained in neuronal growth medium (NGM; CDM supplemented with 2.5% FBS, 20 ng/mL BDNF, 20 ng/mL GDNF, 1 µM cAMP, 100 µM ascorbic acid) containing doxycycline (2.5 µg/mL) and the Notch inhibitor DAPT (2.5 µM; Selleckchem), for 5 days. Neuronal maturation continued in NGM without DAPT for an additional week. Finally, neurons were maintained in BrainPhys™ medium supplemented with 2.5% FBS, B27, N2, GlutaMAX, and penicillin-streptomycin before the experiments.

### In vitro infection and pharmacological treatment

Differentiated neurons (5 × 10⁵ cells/well) were seeded into 12-well plates and inoculated with SARS-CoV-2 at a MOI of 1. After 1 h, the inoculum was removed, the cells were washed twice with PBS, and fresh medium was added.

For orexin rescue experiments, recombinant orexin-A and orexin-B (MCE, HY-106224B, HY-P1339B) were added at the indicated concentrations (10 nM, 100 nM, or 5 µM) immediately after infection. To assess receptor specificity, neurons were pre-treated with the dual orexin receptor antagonist Suvorexant (10 µM; Biorbyt, orb146219) or vehicle (DMSO) for 2 h prior to infection and subjected to orexin treatment. The cells were harvested at 72 h post-infection (hpi) for downstream RNA and protein analyses.

### Quantitative RT-PCR

Brain tissues from K18-hACE2 mice infected with the ancestral SARS-CoV-2 or the Beta variant were generated as described previously by Lee et al. [28]. Total RNA was extracted from tissues using the Maxwell RSC Simply RNA Tissue Kit (AS1340; Promega, Madison, WI, USA) according to the manufacturer’s instructions. Quantitative RT-PCR was performed on a QuantStudio 3 Real-Time PCR System (Applied Biosystems, Foster City, CA, USA) using either the One-Step PrimeScript III RT-qPCR Mix (RR600A) or the One Step TB Green PrimeScript RT-PCR Kit II (RR086A) (Takara, Kyoto, Japan), following the manufacturer’s protocols. Viral RNA targeting the SARS-CoV-2 nucleocapsid (N) gene was detected using the 2019-nCoV-N1 probe (10006770; Integrated DNA Technologies, Coralville, IA, USA). The expression of *Hcrt*, *Rbfox3* and *β-actin* genes was analysed using customised primers (Bionics, Korea). Primer sequences used in this study are listed in Supplementary Table 1. Average threshold cycle (Ct) values of *Hcrt* and *Rbfox3* from the PCRs were normalised to the average Ct values of *β-actin*.

### RNA-seq and analysis

The sequencing library was prepared using the TruSeq Stranded mRNA Sample Prep Kit and sequenced on a NovaSeq 6000 (Illumina, San Diego, CA, USA), yielding more than 6G bases of sequence per sample. Adaptor sequences were removed from the sequenced reads using Trimmomatic (version 0.39) [63] and the processed reads were aligned to the mouse reference genome (GRCm38/mm10) using HISAT2 (version 2.2.1) [64]. The aligned reads were quantified at the gene level using FeatureCounts (version 2.0.6) [65]. Differential expression analyses were performed using DESeq2 (version 1.42.1) [66]. Differentially expressed genes were visualised as volcano plots generated with the EnhancedVolcano package (version 1.20.0) and identified using the Wald test with abs (log_2_ fold change) > 1 and adjusted P-value (Benjamini–Hochberg) < 0.01 as the cut-off. Multidimensional scaling analysis was performed using the cmdscale function in the R stats package (version 4.3.3) based on genes with non-zero expression and non-zero variance among samples. Gene Ontology (GO) enrichment analysis was conducted for DEGs using the enrichGO function in the clusterProfiler package (version 4.10.1) [67], with gene annotations from the org.Mm.eg.db database. Enriched GO terms were defined by P-value < 0.05 and q-value < 0.05. Associations between enriched GO terms and DEGs were represented as networks constructed using igraph (version 2.2.1) [68] and visualised using ggraph (version 2.2.2) in R.

### Multiplex profiling

The brains of SARS-CoV-2-infected mice were dissected at 0, 6, 15, 30, 60, and 90 dpi and homogenised in bead tubes (a-PSBT, GeneReach Biotechnology, Taichung, Taiwan). These homogenates were aliquoted and analysed with the Luminex mouse cytokine/chemokine magnetic bead panel (LXSAMSM-21; LXSAMSM-03, R&D Systems, Minneapolis, MN, USA) using a Bio-Plex 200 multiplexing instrument (Bio-Rad, Hercules, CA, USA) to assess the cytokine/chemokine expression.

### Immunohistochemistry

As previously described [69, 70], mouse brain tissues were coronally prepared at 30 µm thickness from the olfactory bulb to cerebellum. The sections were rinsed in PBS and incubated overnight with mouse anti-NeuN (1:1000; Merck Millipore) and anti-orexin/prepro-orexin (AB3096; Merck Millipore) antibodies for staining general neurons. The next day, the tissue sections were rinsed with 0.5% bovine serum albumin in PBS and incubated for 1 h at room temperature (RT) with a mouse biotinylated secondary antibody (1:400; KPL, MD, USA). This was followed by incubation with an avidin-biotin-peroxidase complex (Vectastain ABC Kit; Vector Laboratories) for visualisation. The bound antiserum was then visualized by incubating with 3,3’ diaminobenzidine (DAB: Sigma) solution under a bright-field microscope (Olympus Optical, Tokyo, Japan).

For Nissl Staining, whole brain sections were mounted on Aminosilane-coated slides and dried for 1 h at RT, dehydrated in 100% ethanol, cleared in xylene, hydrated in a decreasing alcohol gradient, stained with 0.5% cresyl violet (Sigma), washed in distilled water, dehydrated in 100% ethanol, mounted with a coverslip, and viewed under a bright-field microscope (Olympus Optical).

### Immunofluorescence

Immunofluorescence assays were performed as described by Jeong et al. [71]. Briefly, mouse brains were perfused and fixed in 4% paraformaldehyde, sectioned at 30 μm, and subjected to standard permeabilisation and blocking procedures. Sections were incubated with primary antibodies, anti-SARS-CoV-2 nucleocapsid protein (40143-MM05, Sino Biological), and anti-orexin/prepro-orexin (AB3096, Merck Millipore), followed by incubation with Alexa Fluor 488-conjugated anti-rabbit antibody (A32731, Thermo Fisher Scientific, Waltham, MA, USA) and Alexa Fluor 594-conjugated anti-mouse antibody (A32744, Thermo Fisher Scientific). Nuclei were counterstained with DAPI. Immunofluorescence was observed using confocal microscopy (LSM700; Carl Zeiss, Oberkochen, Germany).

### ELISA

Orexin levels were quantified in brain homogenate supernatants prepared as described above. The concentration of each was determined using a Mouse Hcrt(Orexin) ELISA Kit (EM0453, FineTest) according to the manufacturer’s instructions.

### Western blotting

For protein analysis, cultured neurons or brain tissue were lysed in radioimmunoprecipitation (RIPA) buffer (Thermo Fisher Scientific), and proteins in the lysate were separated on a denaturing polyacrylamide gel and transferred to a polyvinylidene fluoride (PVDF) membrane (Merck Millipore, Burlington, MA, USA). The membrane was blocked with 5% skimmed milk (BD Biosciences) in Tris-buffered saline with 0.1% Tween 20 (TBST) buffer and then incubated with the primary antibodies anti-SARS-CoV-2 NP (1:1000, 40143-R001, Sino Biological), anti-NeuN (1:1000, A19086, ABclonal), and anti-β-actin (1:5000, sc-47778, Santa Cruz Biotechnology). Horseradish peroxidase (HRP)-conjugated secondary antibodies (Bio-Rad) and enhanced chemiluminescence (ECL) reagents (Thermo Fisher Scientific) were used for protein band detection. Immunoblot band intensities were quantified by densitometry using ImageJ [72].

### Quantification of cortical NeuN-depleted area and orexin-positive neurons

Brains were coronally sectioned at a thickness of 30 μm using a sliding microtome. Sections were immunostained with antibodies against NeuN or orexin-A/B as described above. To quantify focal NeuN-depleted areas in the cortex, three representative coronal levels were selected from each mouse: anterior cortex (AP: +1.0 mm and 0 mm from bregma) and posterior cortex (AP: −3.4 mm from bregma). The ratio of NeuN-depleted area to total cortical area was measured using ImageJ software (NIH, Bethesda, MD, USA). To quantify orexin-positive neurons in the hypothalamus, stereological cell counting was performed using the optical fractionator method with a bright-field microscope (BX51; Olympus) equipped with Stereo Investigator software (MBF Bioscience), as previously described [73].

### Detection of neuronal death in situ

To assess apoptotic cell death in cortical regions showing reduced NeuN immunoreactivity, the Click-iT™ Plus TUNEL Assay (Thermo Fisher Scientific) was performed on 30-µm-thick brain sections according to the manufacturer’s instructions. Briefly, mounted sections were fixed in 4% paraformaldehyde and permeabilized with Proteinase K for 15 min, followed by a TdT-catalyzed reaction at 37 °C to label fragmented DNA. Apoptotic signals were visualized using Alexa Fluor 488 via a click chemistry-based reaction. After nuclear counterstaining with Hoechst 33,342, sections were imaged using a confocal laser scanning microscope (LSM 900; Carl Zeiss).

### Statistical analysis

Data were analysed using the GraphPad Prism 8.4.3 software (GraphPad Software, San Diego, CA, USA). For multiplex cytokine profiling, values falling below the limit of detection (LOD) were handled based on the analysis type: for heatmap visualisation, undetected values were imputed as 1.0 to facilitate log_2_ transformation (yielding a value of 0). For quantitative comparisons and extended data (Violin plots), these values were assigned as 0 to accurately reflect sample sizes and non-responders. For the multiplex cytokine dataset, outliers were identified separately for each analyte and removed using the ROUT method (Q = 1%). The excluded data points for each analyte are indicated in the RAW DATA file. Unless otherwise stated, no ROUT-based outlier removal was applied to other datasets. Data are presented as the mean ± s.e.m. For comparisons between two groups, an unpaired two-tailed Student’s t-test was used. For comparisons among three or more groups, one-way analysis of variance (ANOVA) with Dunnett’s multiple comparison test was used. Long-term assessment of body weight data was performed using a two-way ANOVA with repeated measures, followed by Sidak’s multiple comparisons test. Survival curves were compared using the log-rank (Mantel–Cox) test. The correlation between viral load and gene expression was assessed using Pearson correlation analysis. A *P-value* < 0.05 was considered statistically significant. The specific statistical tests and sample sizes used are indicated in the figure legends. Schematic overviews for animal experiments were created with BioRender.com.

## Supplementary Information

Supplementary Material 1. Supplementary Fig S1.–S11. and Supplementary Table S1.

Supplementary Material 2.

## Data Availability

The RNA-seq datasets generated in this study have been deposited in the NCBI Sequence Read Archive under accession number PRJNA1430959 and are available to reviewers via the provided review link. Other datasets generated and analyzed during the current study are available from the corresponding author (Y.-C.K.) on reasonable request.
