# Supplementary material for: SARS-CoV-2 infection is associated with hypothalamic orexin suppression and persistent cortical NeuN attenuation
Source: J Neuroinflammation. 2026 May 5;23:216. doi: 10.1186/s12974-026-03842-y (PMC13312555; doi:10.1186/s12974-026-03842-y)
Supplement: Supplementary file 1 — Supplementary Material 1. Supplementary Fig S1.–S11. and Supplementary Table S1. [file 12974_2026_3842_MOESM1_ESM.docx]

**Supplementary data**

**
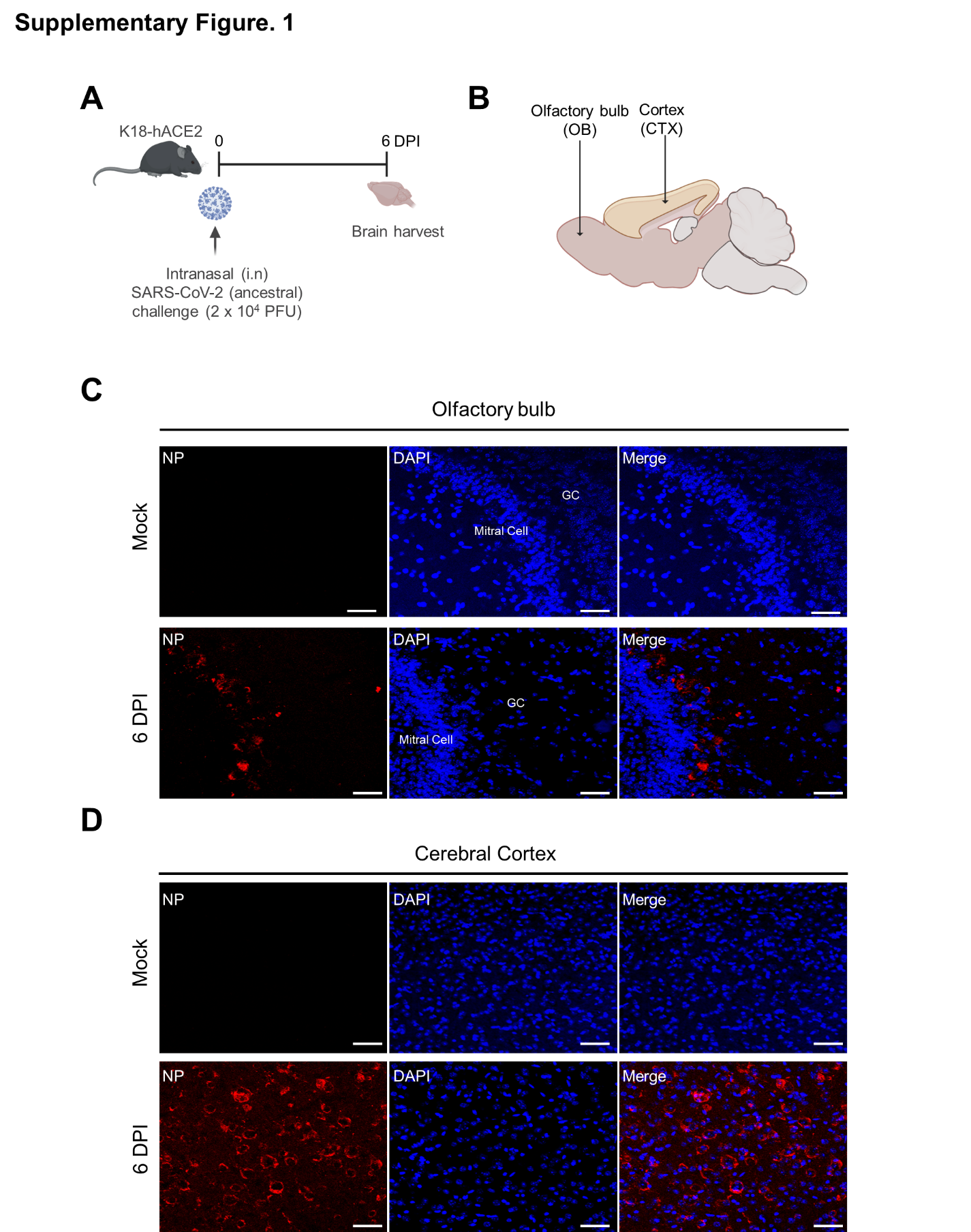
**

**Supplementary Figure 1. Extensive brain infection and neuroinvasion in K18-hACE2 transgenic mice infected with a lethal dose of SARS-CoV-2.**

(A) Schematic of the lethal-dose infection model. K18-hACE2 mice were intranasally challenged with 2 × 10^4^ PFU of SARS-CoV-2 and analysed at 6 dpi. (B) Diagram of the mouse brain indicating the anatomical regions analysed for viral distribution: olfactory bulb (OB), cortex (CTX). (C-D) Representative immunofluorescence images of the SARS-CoV-2 nucleocapsid (NP; red) in the olfactory region (C) and cerebral cortex (D) at 6 dpi. Nuclei are counterstained with DAPI (blue) and merged images are shown. Scale bars, 25 µm. Images are representative of three n = 3 mice.


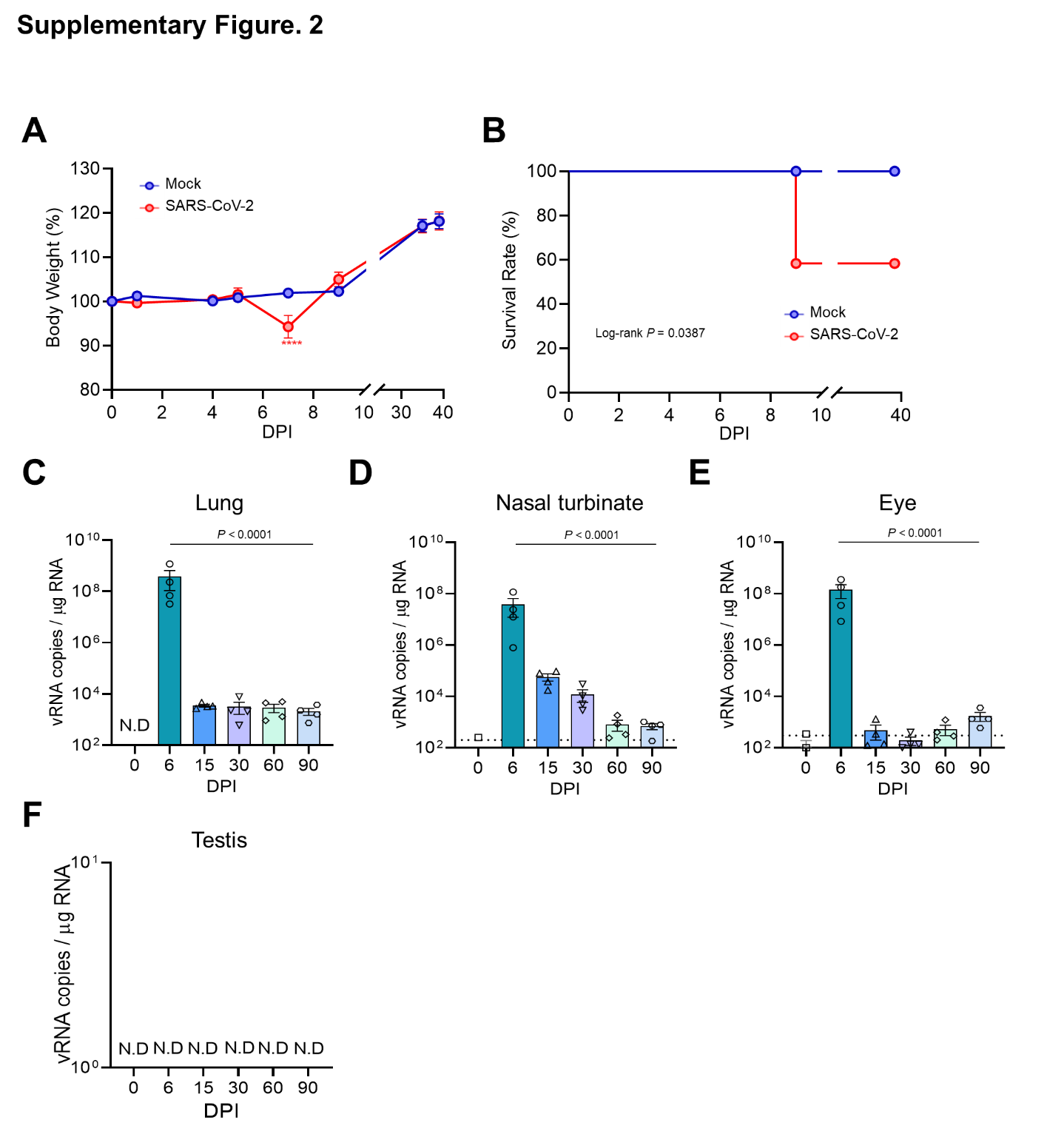


**Supplementary Figure 2. Clinical course and peripheral viral RNA kinetics following SARS-CoV-2 infection in K18-hACE2 mice.**

(A-B) Body weight change (A) (n = 12 mice per group) and survival (B) (n = 13 mice per group) of K18-hACE2 mice infected intranasally with 50 PFU of SARS-CoV-2 compared with mock controls. (C-F) Time-course quantification of viral RNA in the lung (C), nasal turbinate (D), eye (E) and testis (F) at the indicated time points using RT-qPCR (n = 4 mice per group per time point). Limits of detection (LOD) indicated by the dotted lines. The dashed line indicates the limit of detection; viral RNA in the testis was not detected at any time point. N.D., not detected. Data are the mean ± s.e.m. Statistical significance was determined by two-way ANOVA with repeated measures followed by Sidak’s multiple-comparisons test (A), log-rank (Mantel–Cox) test (B), and one-way ANOVA with Dunnett’s multiple-comparisons test (C-E). The exact *P* values are indicated; **** *P* < 0.0001.


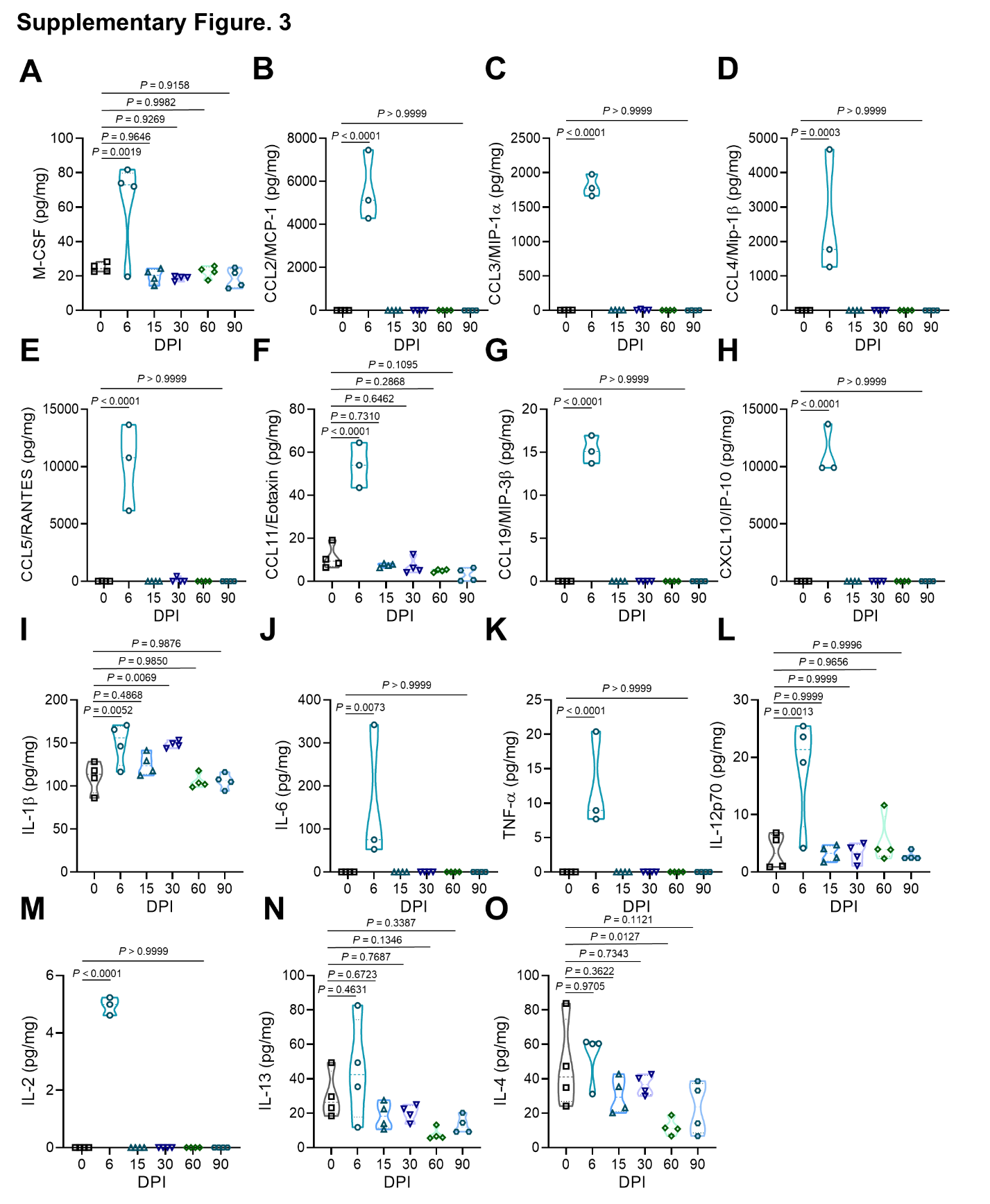


**Supplementary Figure 3. Multiplex profiling of cytokines and chemokines in whole-brain lysates from SARS-CoV-2-infected K18-hACE2 mice.**

(A-O) Cytokine and chemokine protein concentrations measured in whole-brain lysates at 0 (mock), 6, 15, 30, 60 and 90 dpi using a multiplex bead-based array. Analytes include M-CSF (A), CCL2 (B), CCL3 (C), CCL4 (D), CCL5 (E), CCL11 (F), CCL19 (G), CXCL10 (H), IL-1β (I), IL-6 (J), TNF-α (K), IL-12p70 (L), IL-2 (M), IL-13 (N), and IL-4 (O). The data are presented as violin plots with overlaid individual data points (n = 4 per group at each time point; sporadic statistical outliers were excluded using the ROUT method, detailed in Methods). Non-detected samples are assigned a value of 0. Statistical significance was determined using one-way analysis of variance (ANOVA) with Dunnett’s multiple-comparisons test (each time point compared with 0 dpi). The exact *P* values are indicated.


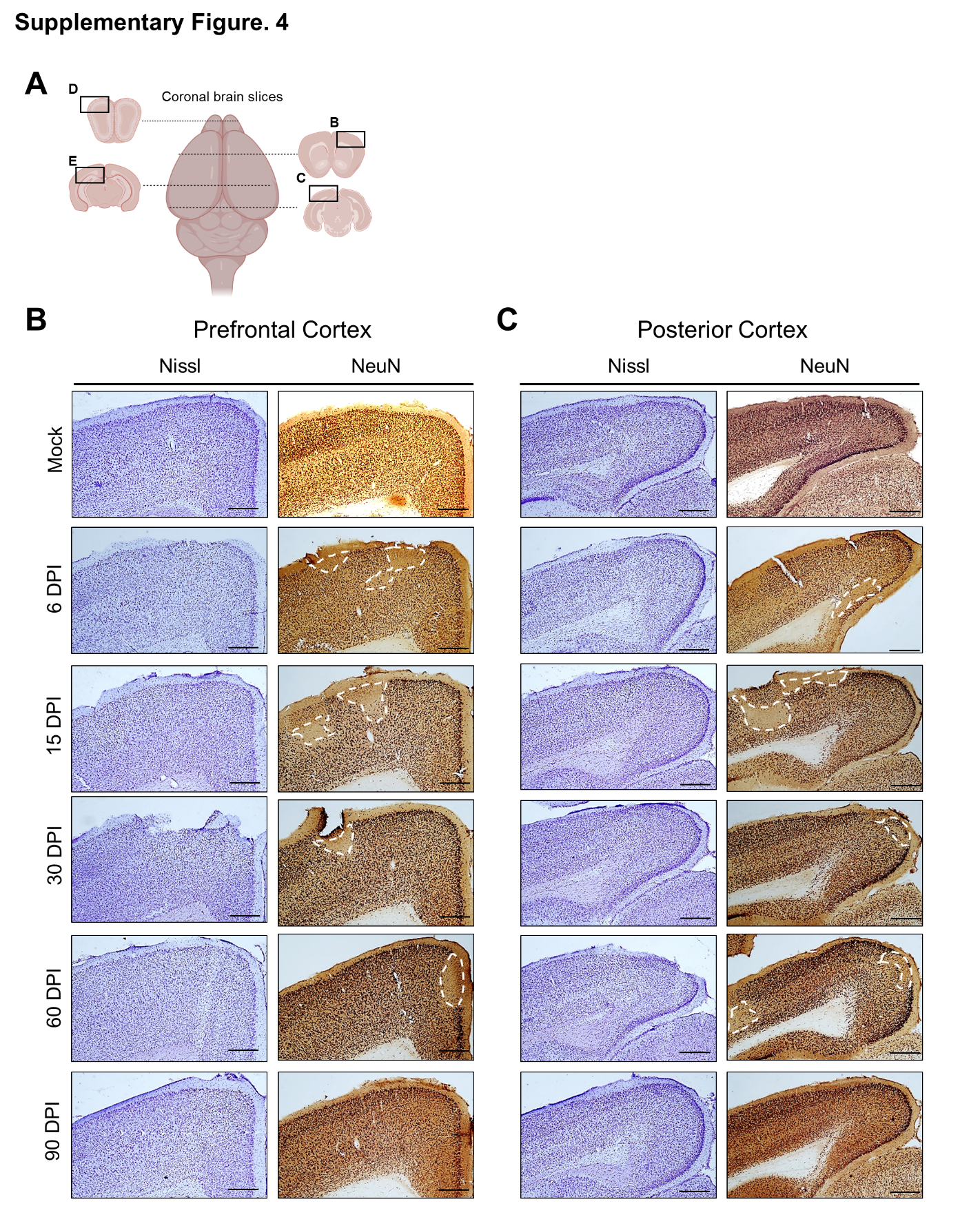


**
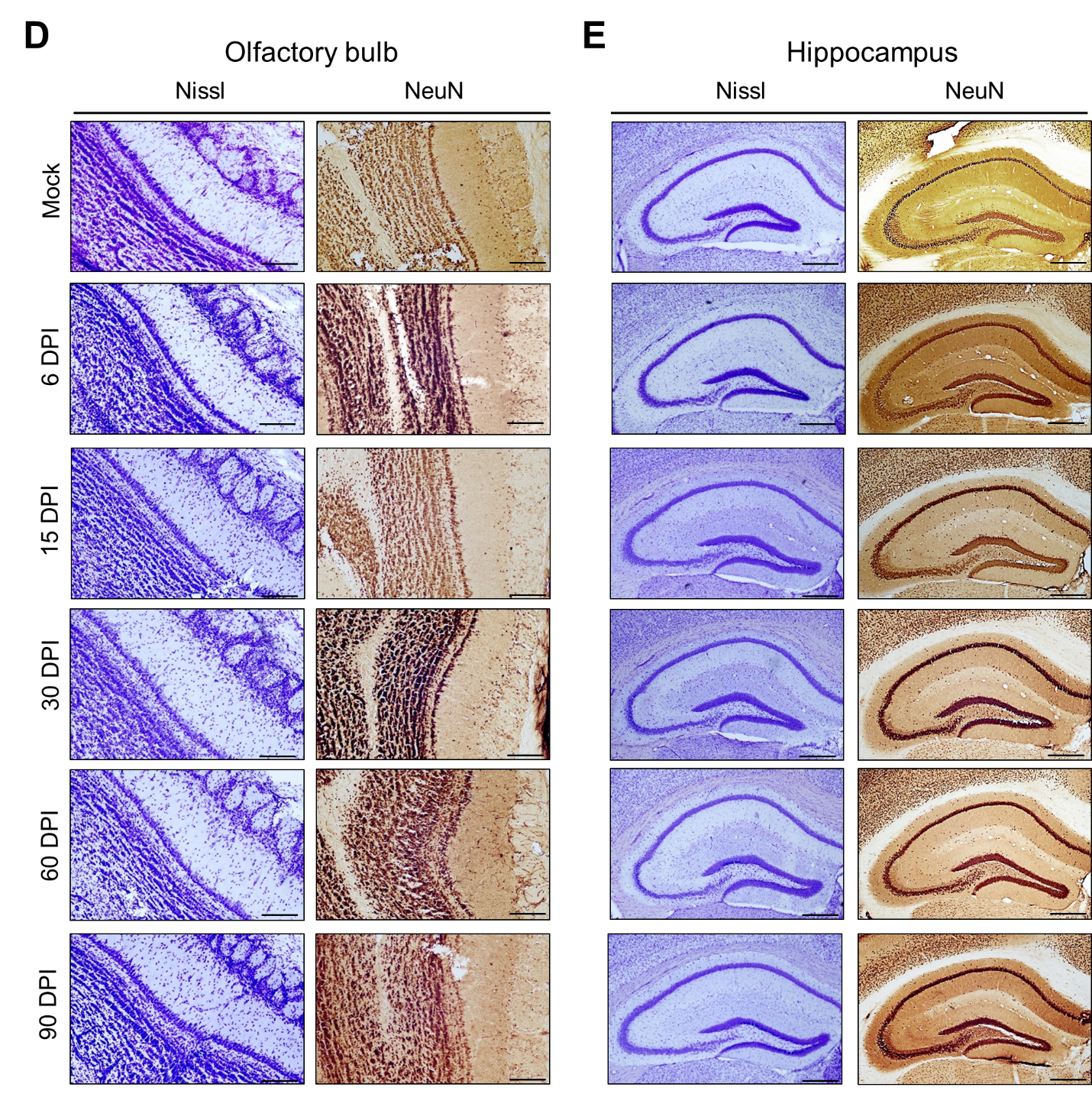
**

**Supplementary Figure 4. Persistent patchy reduction of neuronal NeuN expression in the cortex of K18-hACE2 mice following SARS-CoV-2 infection.**

**(A)** Schematic of coronal brain sections indicating the anatomical locations of the prefrontal cortex (B), posterior cortex (C), olfactory bulb (D) and hippocampus (E). (B-E) Representative images of Nissl (left) and NeuN (right) staining in the indicated brain regions from mock-infected and SARS-CoV-2-infected mice at 6, 15, 30, 60, and 90 dpi. White dashed lines demarcate focal areas of reduced NeuN immunoreactivity observed in the cortical regions (B-C), in contrast to preserved staining in the olfactory bulb (D) and hippocampus (E). Scale bars, 100 µm. The images are representative of n = 3-5 mice with similar results.


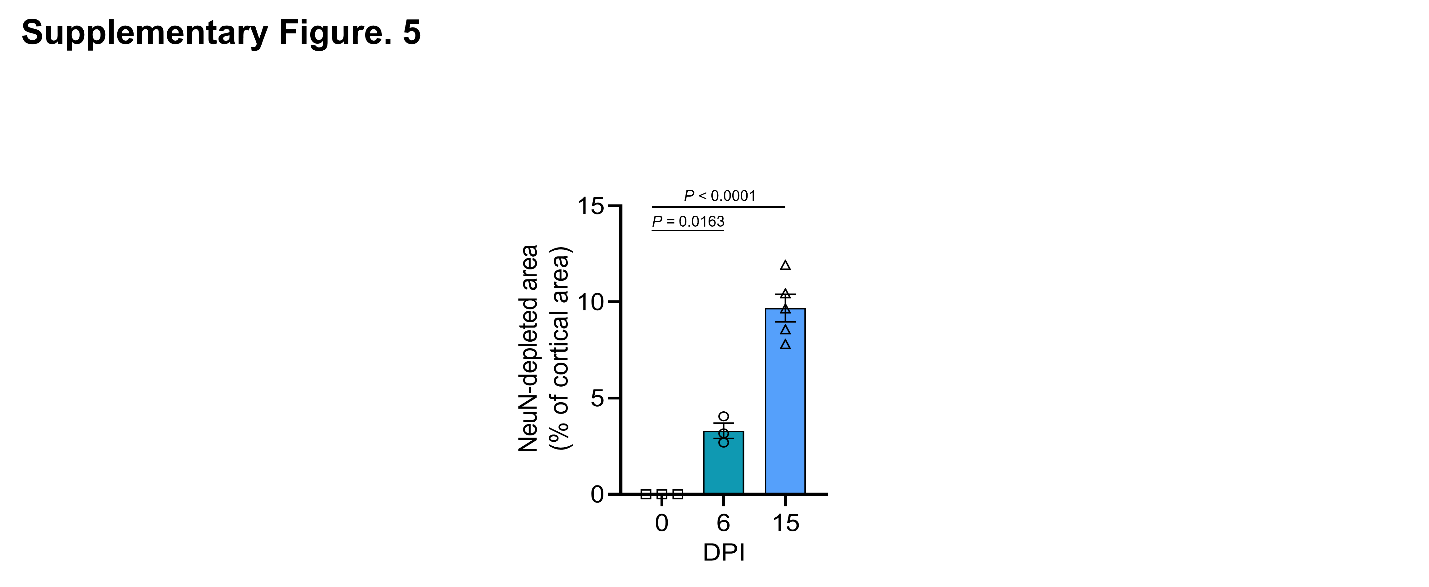


**Supplementary Figure 5. Semi-quantitative area analysis of focal cortical NeuN-depleted regions in K18-hACE2 mice.**

The representative cortical NeuN-low regions shown in Fig. 1E were assessed by measuring the NeuN-depleted area as a percentage of total cortical area at the indicated days post infection (dpi). Individual symbols represent biologically independent animals. Data are shown as mean ± s.e.m. Statistical significance was determined by one-way ANOVA with Dunnett’s multiple-comparisons test. The exact *P* values are indicated.

**
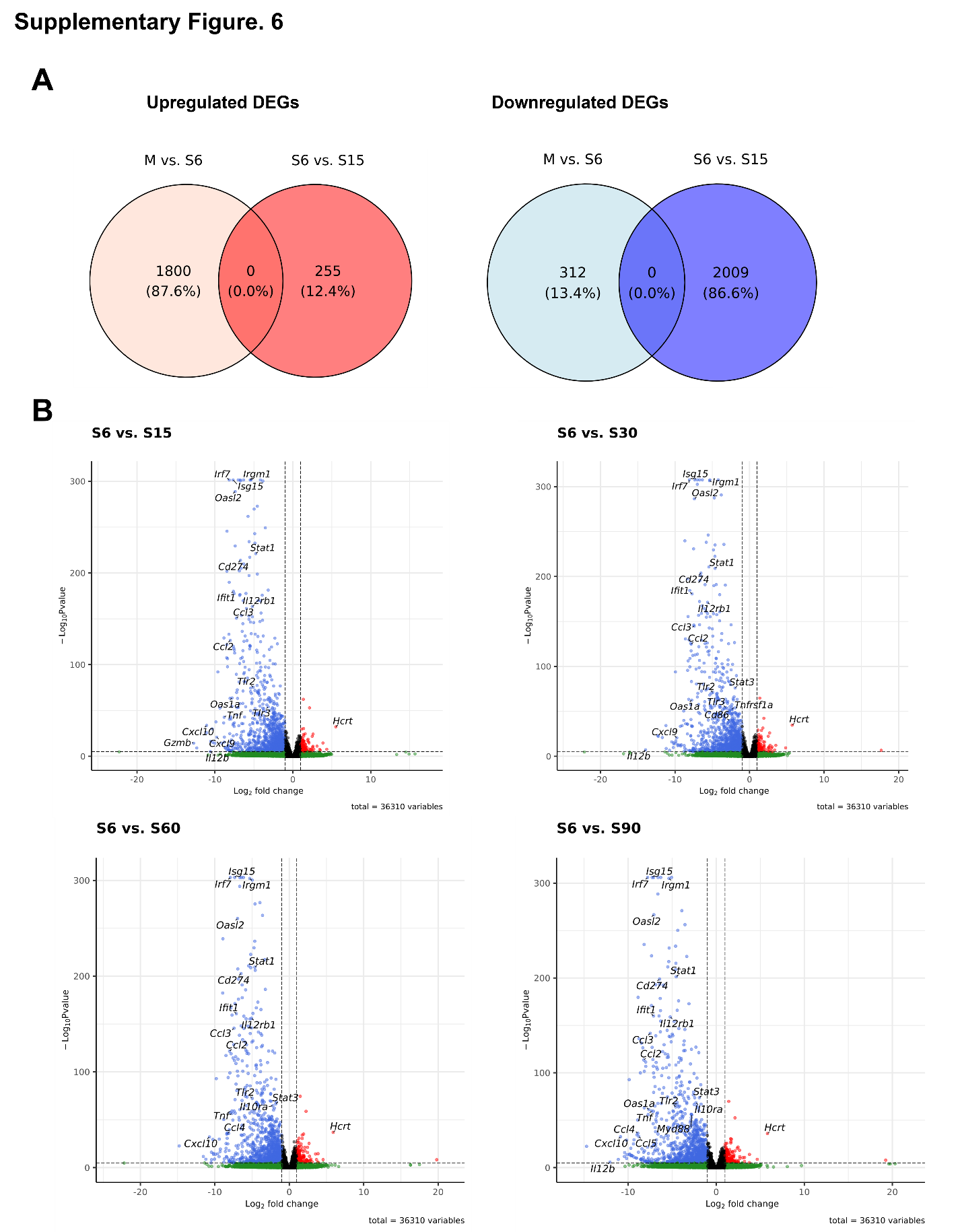
Supplementary Figure 6. Transcriptomic profiling of SARS-CoV-2-infected K18-hACE2 mouse brains using RNA-seq.**

(A) Venn diagrams illustrating the overlap of upregulated (left) and downregulated (right) differentially expressed genes (DEGs) between mock versus 6 days post-infection (dpi) (M vs. S6), and 6 dpi versus 15 dpi (S6 vs. S15) (M, mock; S6, 6 dpi; S15, 15 dpi). (B) Volcano plots depicting differentially expressed genes (DEGs) for pairwise comparisons: 6 dpi versus 15 dpi, 6 dpi versus 30 dpi, 6 dpi versus 60 dpi, and 6 dpi versus 90 dpi. Key immune-related genes and Hcrt are labelled. Blue and red dots represent significantly downregulated and upregulated genes, respectively (log_2_ fold change > 1 or < −1; adjusted *P* < 0.05). The vertical dashed lines indicate the log_2_ fold-change cutoff, while the horizontal dashed line indicates the significance threshold.


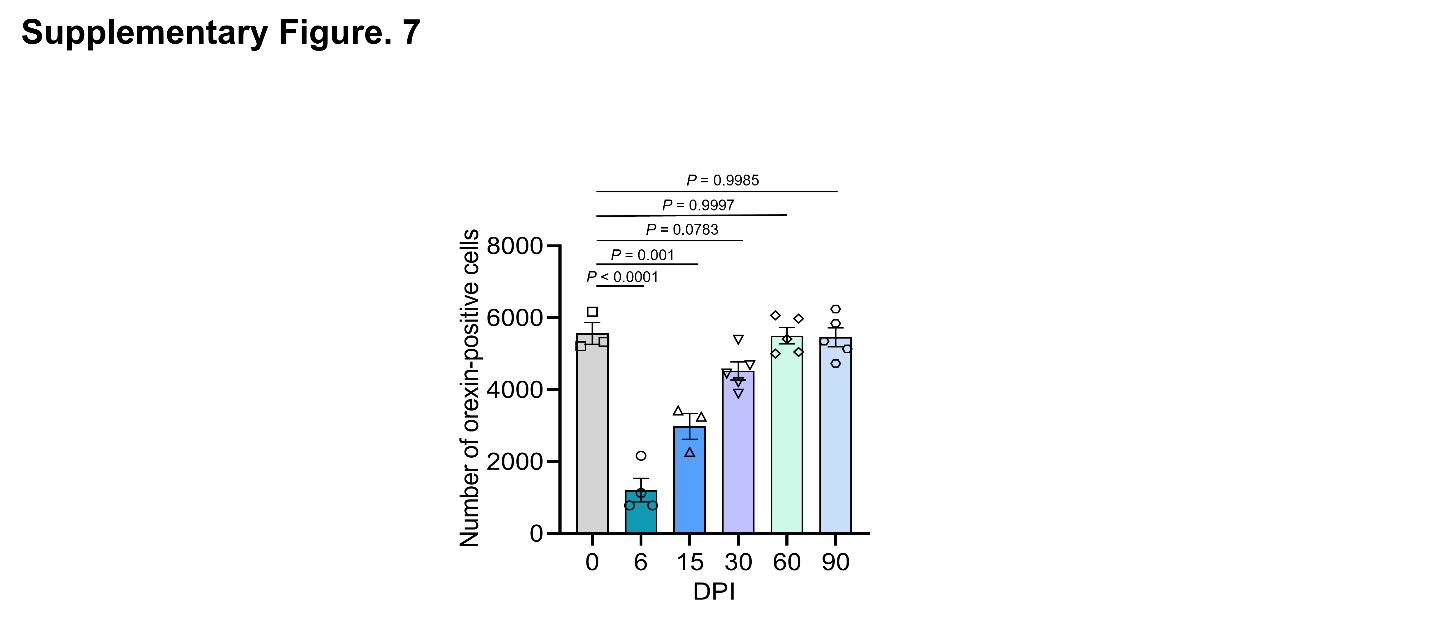
**Supplementary Figure 7. Semi-quantitative counting of orexin-positive cells in the lateral hypothalamus of K18-hACE2 mice following SARS-CoV-2 infection.**

Orexin-positive cells were counted in the lateral hypothalamus at the indicated days post infection (dpi). The quantification supports the qualitative reduction in orexin immunoreactivity observed at 6 dpi, followed by recovery at later time points. Individual symbols represent biologically independent animals. Data are shown as mean ± s.e.m. Statistical significance was determined by one-way ANOVA with Dunnett’s multiple-comparisons test. The exact *P* values are indicated.

**
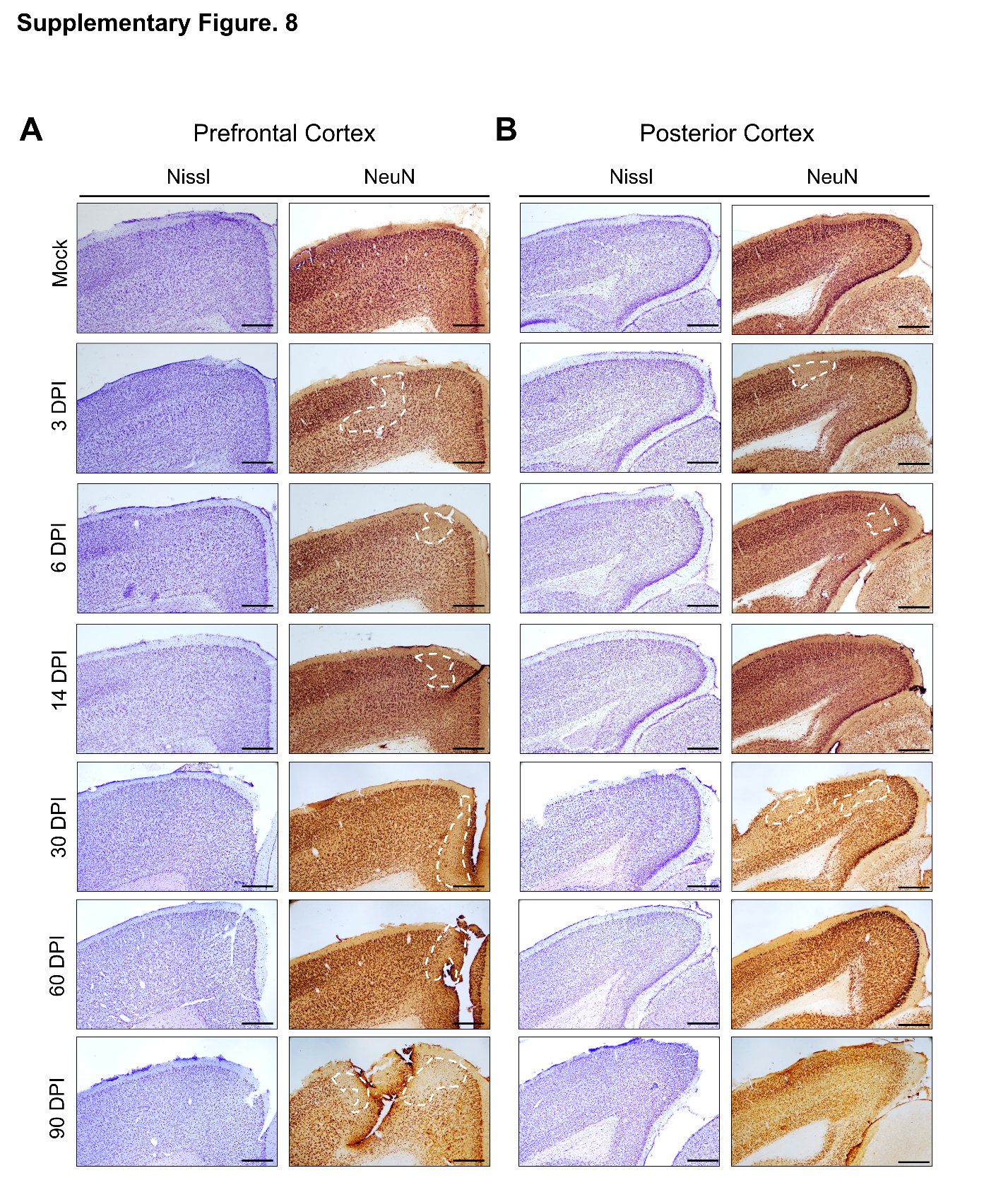

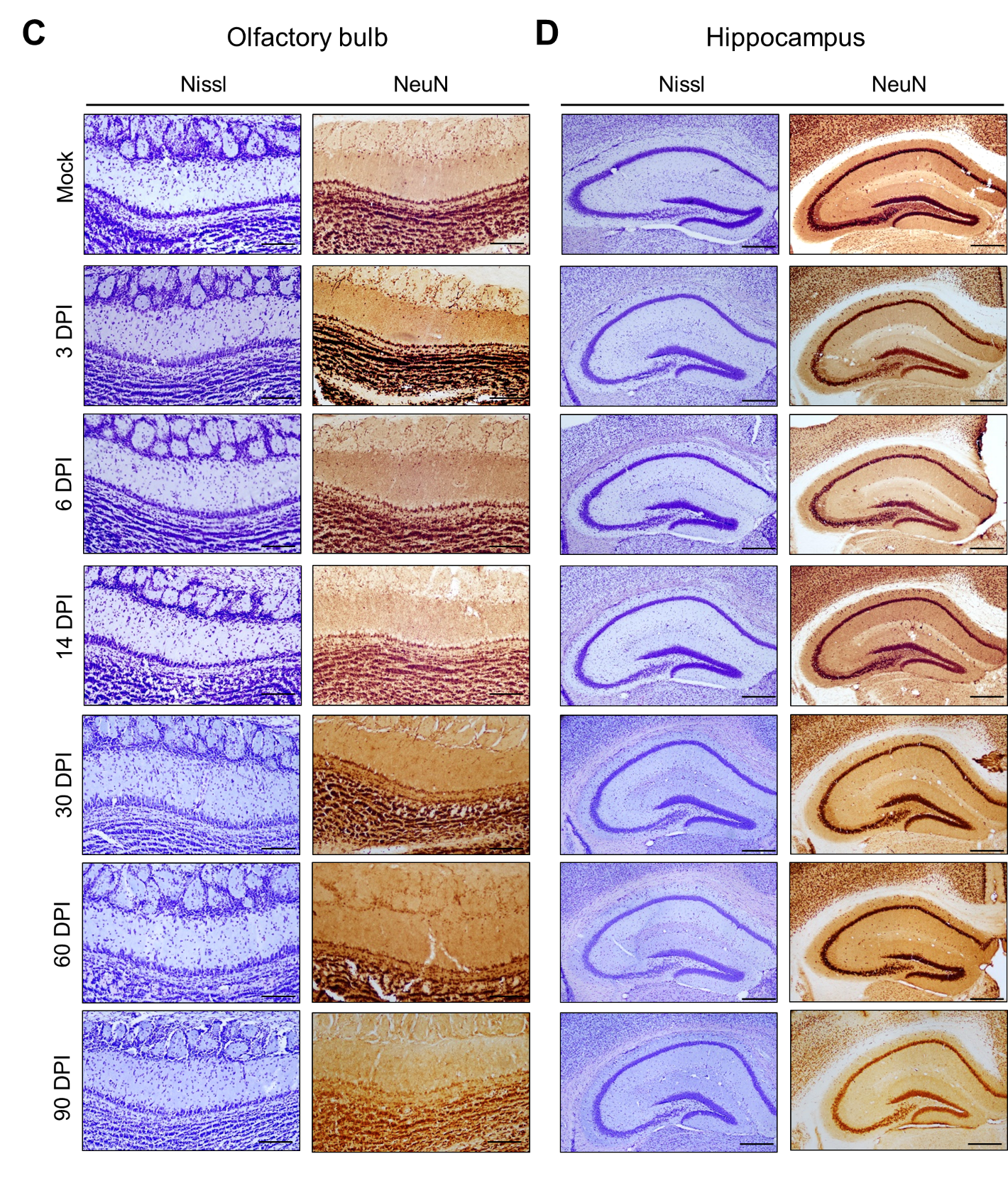
**

**Supplementary Figure 8. Persistent patchy reduction of cortical NeuN immunoreactivity in BALB/c mice following MA10 SARS-CoV-2 infection.**

(A-B) Representative images of Nissl staining (left) and NeuN immunohistochemistry (right) of the prefrontal cortex (A) and posterior cortex (B) of mock- and MA10-infected BALB/c mice at 3, 6, 14, 30, 60, and 90 dpi. White dashed lines indicate the focal regions with reduced NeuN immunoreactivity. (C-D) Representative images of Nissl staining and NeuN immunoreactivity in the olfactory bulb (C) and hippocampus (D) showing no overt reduction in NeuN immunoreactivity across the analysed time points. Scale bars: 100 µm (all panels). Images are representative of n = 3-4 independent biological replicates with similar results.

**
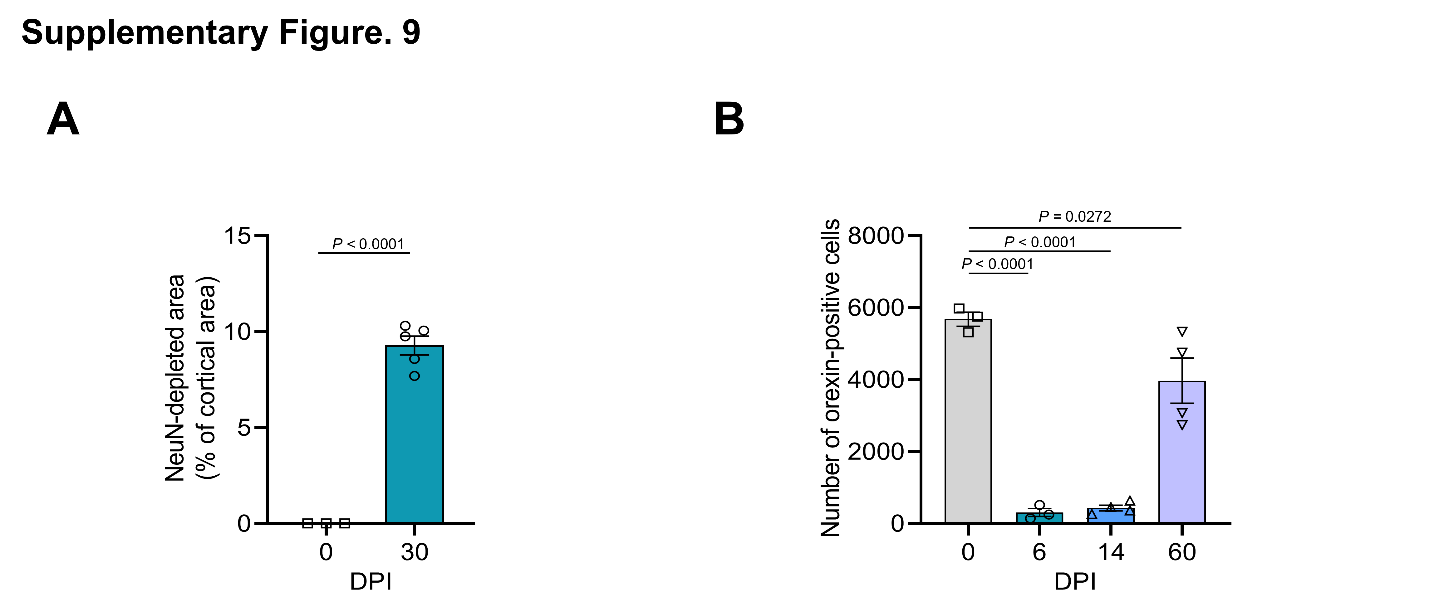
**

**Supplementary Figure 9. Semi-quantitative analysis of cortical NeuN-depleted regions and hypothalamic orexin-positive cells in MA10-infected mice.**

(A) Semi-quantitative area analysis of the representative focal cortical NeuN-depleted regions shown in Fig. 5G. The NeuN-depleted area was measured and expressed as a percentage of total cortical area at the indicated days post infection (dpi). (B) Semi-quantitative counting of orexin-positive cells in the lateral hypothalamus at the indicated dpi following MA10 infection. Individual symbols represent biologically independent animals. Data are shown as mean ± s.e.m. Statistical significance was determined using a two-tailed unpaired Student’s t-test (A) or one-way ANOVA with Dunnett’s multiple comparison test (B). The exact *P* values are indicated.


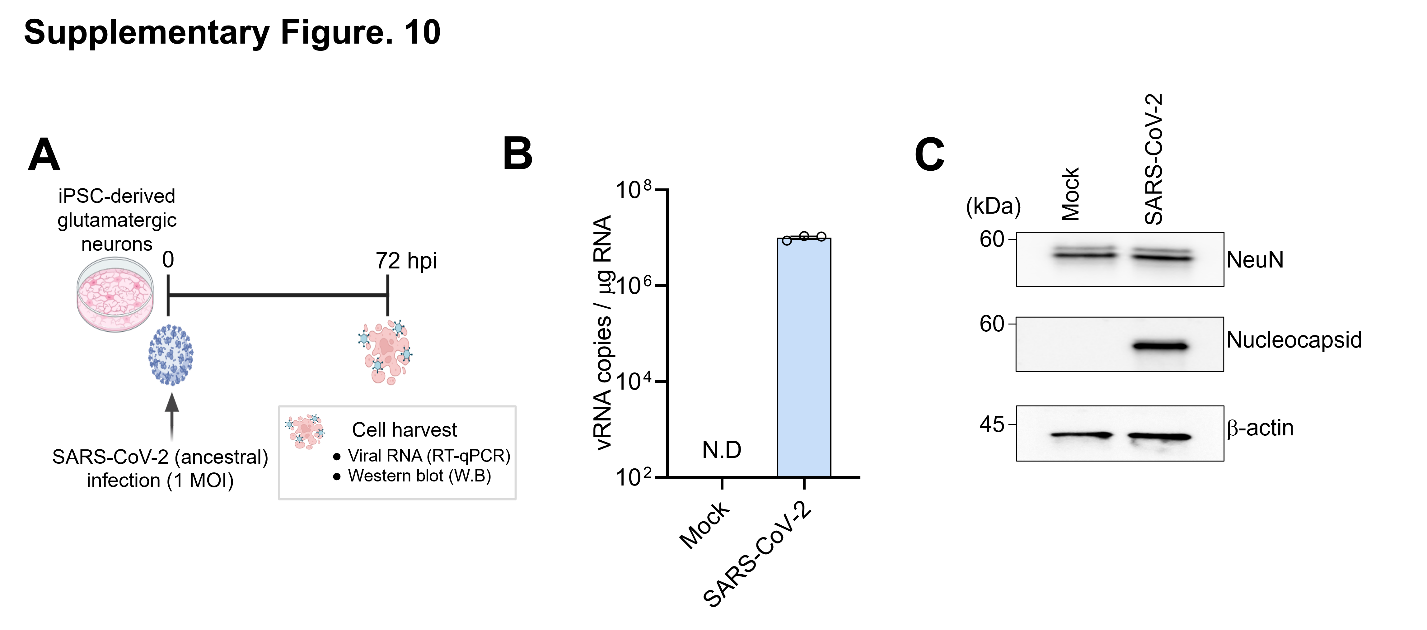


**Supplementary Figure 10. SARS-CoV-2 infection permits robust viral replication but does not deplete NeuN protein in human iPSC-derived glutamatergic neurons.**

(A) Schematic of the experimental timeline. Human iPSC-derived glutamatergic neurons were infected with SARS-CoV-2 at a multiplicity of infection (MOI) of 1. Cells were harvested at 72 h post-infection (hpi) for analysis. (B) Quantification of intracellular SARS-CoV-2 viral RNA loads using RT–qPCR at 72 hpi. Data are presented as viral RNA copies per µg of total RNA (n = 3 independent biological replicates). (C) Western blot analysis of NeuN and SARS-CoV-2 Nucleocapsid (NP) protein expression in mock and SARS-CoV-2-infected neurons at 72 hpi. β-Actin was used as a loading control. Representative immunoblots are shown. Data represent the mean ± s.e.m.

**
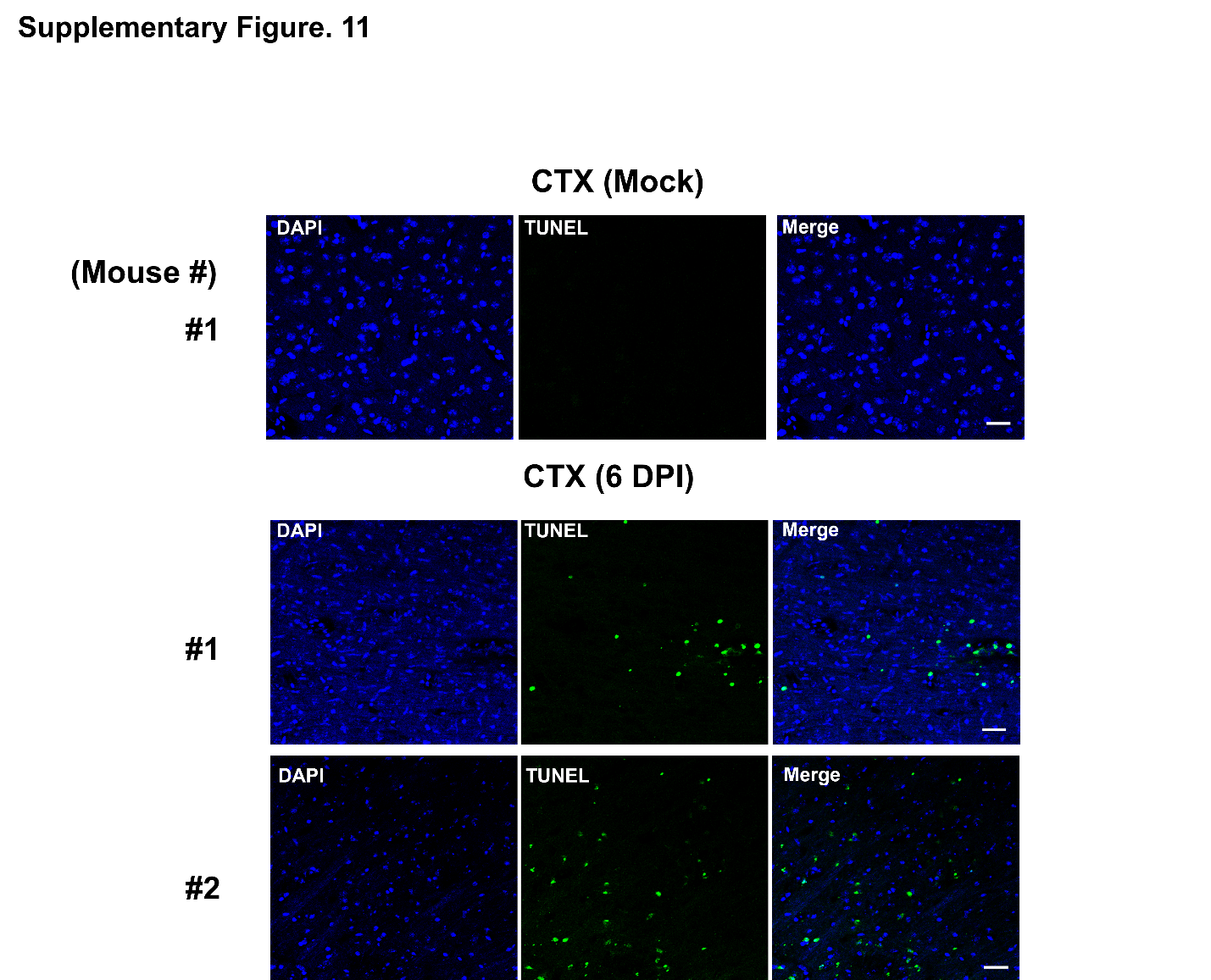
**

**Supplementary Figure 11. Representative cortical TUNEL staining in K18-hACE2 mice infected with SARS-CoV-2.**

DAPI, TUNEL, and merged images are shown for mock and infected cortical sections at 6 dpi after intranasal infection with 2 × 10^4 PFU SARS-CoV-2. Limited TUNEL-positive cells were detected in infected cortex. Scale bars, 25 μm.

**Supplementary Table 1. List of primers used for RT-qPCR.**

| Gene | Forward Primer (5'->3') | Reverse Primer (5'->3') |
| --- | --- | --- |
| *Hcrt* (Mouse) | TGC CGT CTC TAC GAA CTG TTG C | AGC TGC GTG GTT ACC GTT GGC |
| *Rbfox3* (Mouse) | CAC CAC TCT CTT GTC CGT TTG C | GGC TGA GCA TAT CTG TAA GCT GC |
| β-actin(Mouse) | CAT TGC TGA CAG GAT GCA GAA GG | TGC TGG AAG GTG GAC AGT GAG G |
