# Supplementary material for: SARS-CoV-2 infection is associated with hypothalamic orexin suppression and persistent cortical NeuN attenuation
Source: J Neuroinflammation. 2026 May 5;23:216. doi: 10.1186/s12974-026-03842-y (PMC13312555; doi:10.1186/s12974-026-03842-y)

Figure. 7B

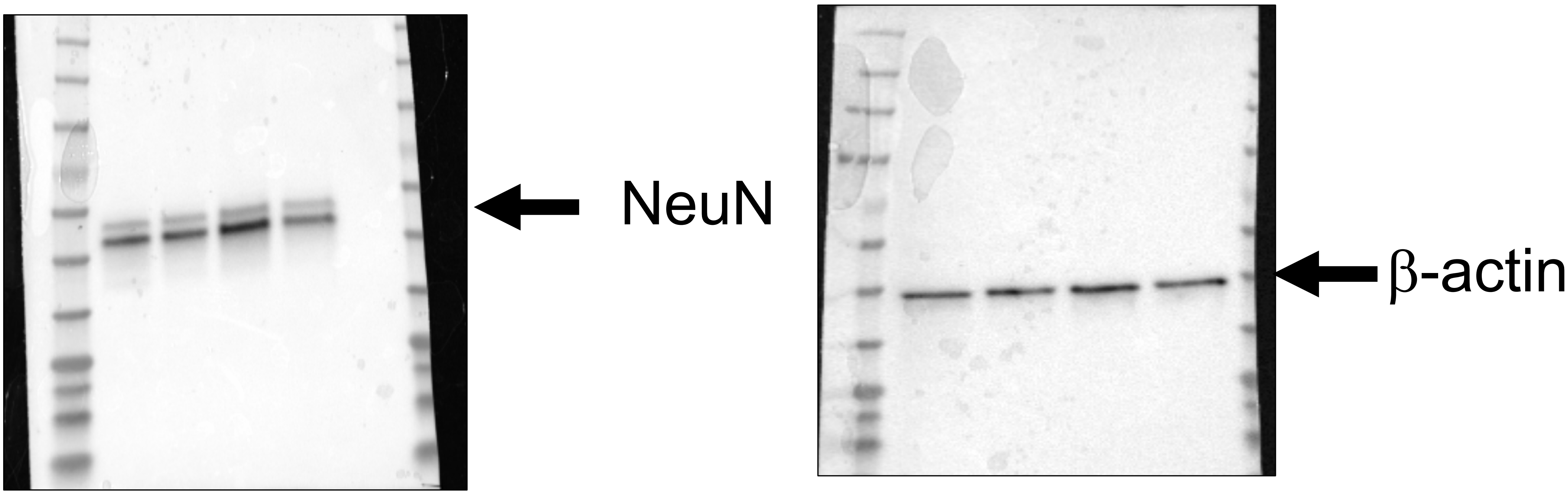

Figure. 7C

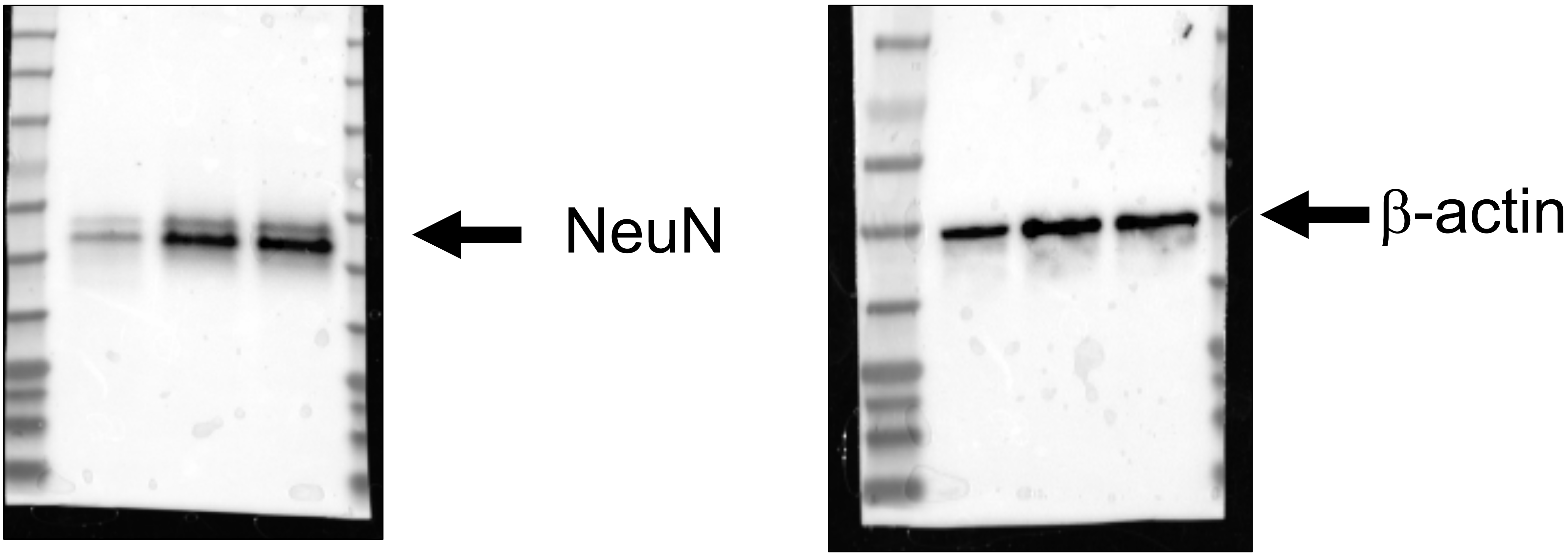

Figure. 7H

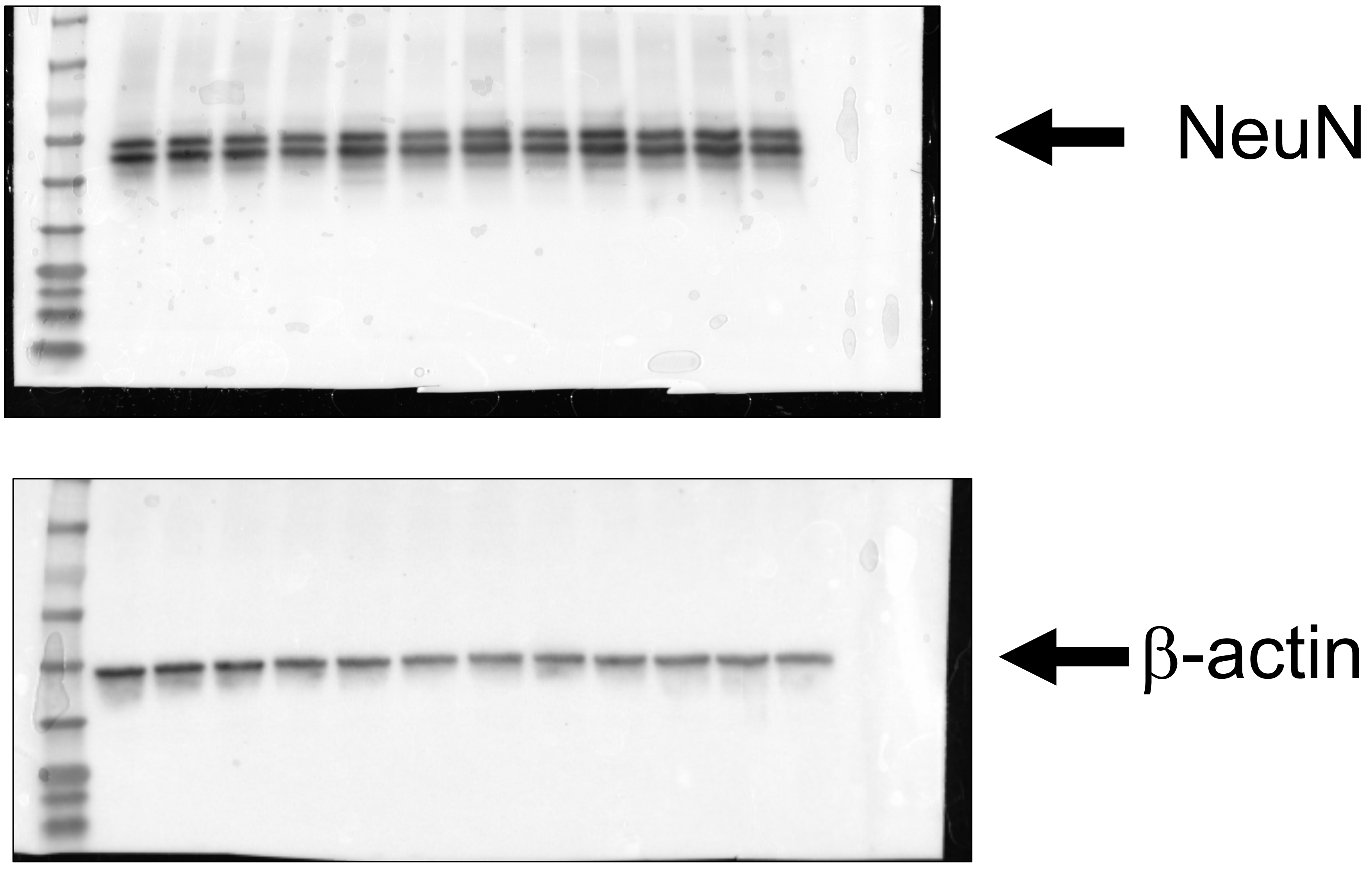

Supplementary Figure. 10C

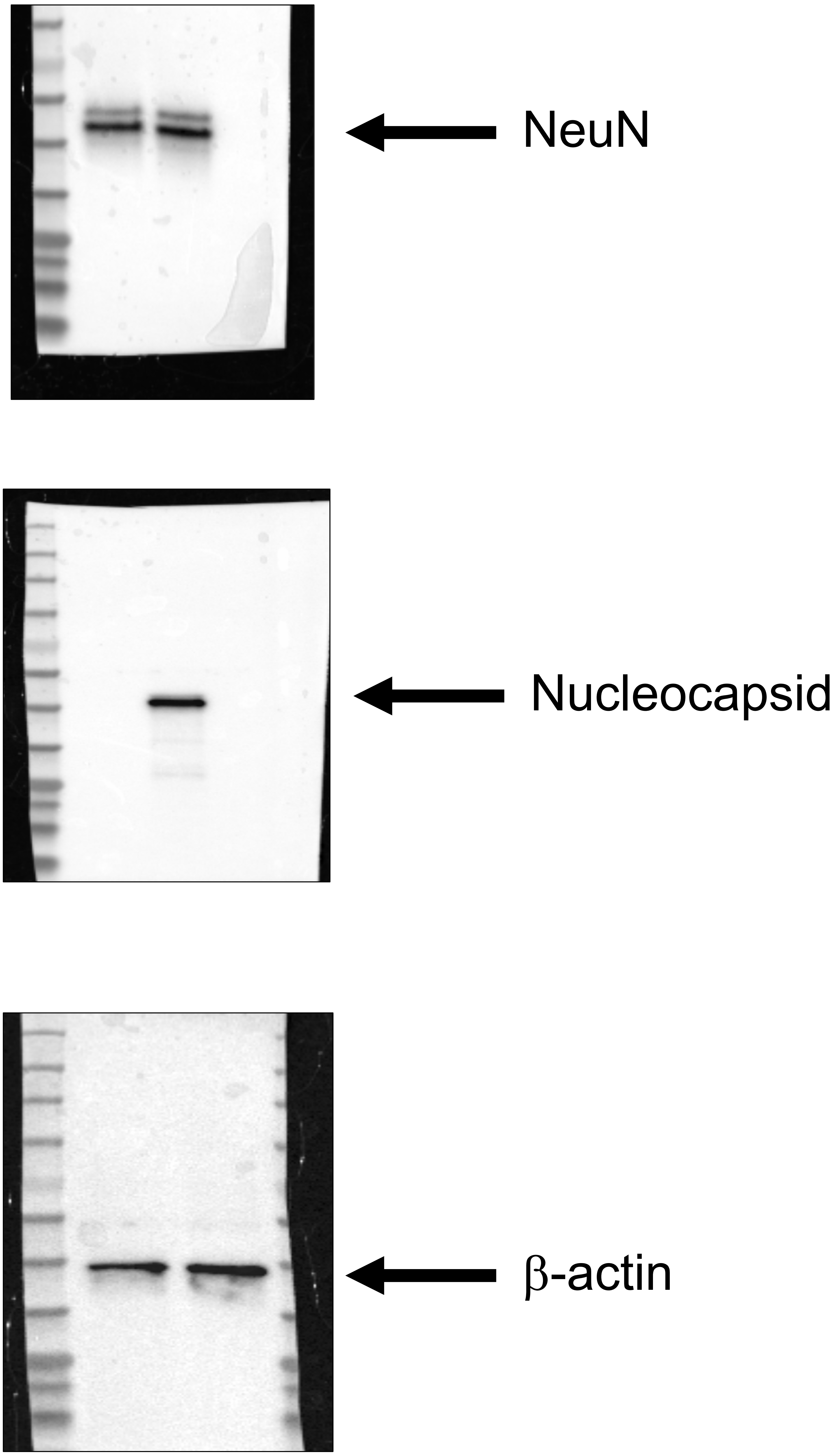

Supplement: Supplementary file 2 — Supplementary Material 2. [file 12974_2026_3842_MOESM2_ESM.pdf]
